# The influence of environmental exposures during the preconception period on offspring outcomes: a systematic review

**DOI:** 10.3389/fpubh.2025.1633266

**Published:** 2025-12-17

**Authors:** Amie Steel, Wen Ray Lee, Zhiwei Xu, Jenny Carè, Javier Cortes-Ramirez, Carrie Thomson-Casey, Dwan Vilcins

**Affiliations:** 1 Faculty of Health, School of Public Health, University of Technology Sydney, Ultimo/Sydney, NSW, Australia; 2 Child Health Research Centre, The University of Queensland, Brisbane, QLD, Australia; 3 School of Medicine and Dentistry, Griffith University, Gold Coast, QLD, Australia; 4 Centre for Data Science, Queensland University of Technology, Brisbane, QLD, Australia

**Keywords:** environmental hazards, preconception, birth outcome, neonatal outcomes, maternal health, family medicine

## Abstract

**Introduction:**

Health behaviors and exposures to environmental hazards among individuals of reproductive age prior to pregnancy can influence maternal and child health outcomes. While research attention has focused on preconception health behaviors, such as diet and lifestyle, there is emerging evidence that environmental exposures may also be important to consider.

**Methods:**

A search strategy (PROSPERO # CRD42021240069) was developed for MEDLINE (OVID), EMBASE (OVID), Maternity and Infant Care (OVID), CINAHL (EBSCO), and PsycINFO (EBSCO). Searches were conducted from database inception until 21 May 2021. Studies were included that investigated male or female exposure to any environmental hazard during the preconception period and reported neonatal or child health outcomes. No limit to date of publication, language or comparator were applied. Studies were critically appraised using the Newcastle-Ottawa Quality Assessment Scale for Cohort studies.

**Results:**

The review identified 63 studies that met the inclusion criteria, published between 1974 and 2021. They encompassed studies that covered ambient exposures (*n* = 23), chemical exposures (*n* = 26), and other exposures (*n* = 24). For ambient exposures, all studies examined the outcomes associated with air pollution and one study also explored associations with exposure to hot and cold ambient temperature. Studies investigating chemical exposures encompassed endocrine-disrupting chemicals (*n* = 4), pesticides (*n* = 10), persistent organic pollutants (*n* = 4), and organic solvents (*n* = 7). Other exposures studied were categorized as radiation (*n* = 9), metals (*n* = 4) and undifferentiated products or compounds (*n* = 14). Outcomes measured by the included studies covered congenital malformations, adverse birth outcomes and childhood illness. There was a high level of heterogeneity across the included studies that precluded meta-analysis. Various associations between exposures and outcomes were identified.

**Discussion:**

There is growing evidence of adverse outcomes in offspring associated with maternal and paternal environmental exposures during the pre-conception period. While there are some topics that have received focused attention from research teams in the last 50 years, most studies appear to be standalone and have not continued to develop as part of wider research programs. There is need to develop a field-wide approach to create an agenda for environmental preconception health exposures and outcomes that supports more coordinated, targeted and strategic research efforts.

**Systematic review registration:**

https://www.crd.york.ac.uk/PROSPERO/view/CRD42021240069

## 1 Introduction

Health behaviors and exposures to environmental hazards among individuals of reproductive age prior to pregnancy can influence maternal, birth and offspring outcomes (1–4). There is a time before a couple become pregnant or plan to become pregnant, that is referred to as the “preconception period” (2). This period—spanning from months to years prior to pregnancy—is critical in terms of the health of the future child and their health across their life course. From a public health perspective, identifying modifiable preconception risk factors and avoiding them could be an opportunity to improve maternal, paternal and child health (5). While research attention has focused on preconception health behaviors, such as diet and lifestyle, there is evidence that environmental exposures—external factors in our environments—are also important considerations (6, 7).

The World Health Organization (WHO) estimates that 23% of global deaths and 24% of disease burden is due to adverse environmental exposures that are not modifiable by health behaviors (8). Common examples include environmental tobacco smoke, air pollutants from vehicle exhaust fumes and industrial manufacturing, pesticides, heavy metals, plasticizers, and flame retardants (7). The United States Centers for Disease Control and Prevention has reported that more than 400 environmental chemicals or their metabolites have been found in human samples including urine, blood, serum, and breast milk (9). Further, a range of environmental hazards are able to cross the placenta (10, 11), and directly expose the developing fetus through diverse transport mechanisms (10). Environmental chemicals (10), heavy metals and black carbon particles (11), for example, have been found on the fetal side of the placenta (10). These environmental exposures can induce adverse effects. Particulate matter has been shown to induce inflammation in the placenta, with preclinical models demonstrating exposure prior to pregnancy causes a decrease in placental mass, size and surface area, even if exposure is not continued through pregnancy (12). Exposure to environmental hazards in the preconception period may induce epigenetic changes in both maternal and paternal germ cells (13–15).

Such preclinical evidence highlights the importance of understanding the real-world outcomes associated with preconception environmental exposures. Perinatal conditions—including prematurity, low birth weight and congenital anomalies—and childhood cluster diseases, are the seventh and the ninth largest contributors, respectively, to death and disease burden due to environmental factors (8). These factors include maternal environmental and occupational exposures to pesticides and other chemicals (8). However, these trends do not meaningfully differentiate between exposure prior to conception and exposure during pregnancy. While previous research has highlighted the diversity of environmental hazards associated with adverse child outcomes after maternal exposure preceding pregnancy (16), there is yet to be a comprehensive review of original research explicitly examining the implications of environmental exposures occurring in the preconception period on birth and child health outcomes. In this context, this systematic review aimed to critically review the current research evidence describing environmental exposures prior to conception associated or correlated with positive or adverse offspring outcomes.

## 2 Materials and methods

### 2.1 Search method

A systematic review protocol was developed following the PRIMSA-P guidelines (1) and registered with PROSPERO (CRD42021240069). A search strategy was developed for MEDLINE (OVID), EMBASE (OVID), Maternity and Infant Care (OVID), CINAHL (EBSCO), and PsycINFO (EBSCO). Searches were conducted from database inception until 21 May 2021 using Boolean operators appropriate to each database (see Supplementary Table 1 for example search strategy). Employing the snowballing technique, the reference lists of related systematic reviews as well as the reference lists and citation trail of articles identified for inclusion in the review were hand searched for relevant studies that were not identified through the electronic searches. There was no limit on date.

Two authors conducted the database searches and the search results were imported into Covidence systematic review software (97).

#### 2.1.1 Selection criteria

The population of interest was reproductive-aged males or females that were self- or researcher-identified as being in the preconception period and as being the reproductive parent of the child under study. Studies were sought that included exposure to any environmental hazard and measured neonatal outcomes or child health outcomes in later life. Environmental exposures were defined as hazards transmitted through environmental media and not managed by health modalities or behavior change (e.g., maternal smoking). The neonatal (>22 weeks completed gestation) outcomes of interest were stillbirth, birthweight, birth length, head circumference, gestational age, and preterm birth.

Articles were excluded if the environmental exposure could not be clearly attributed to the preconception period. This included studies that examined the peri-conceptual period (3–12 mths prior to end first trimester) without differentiating data from before conception. Studies were excluded if the outcomes were not related to birth, neonatal outcomes or child health (i.e., fertility or maternal health outcomes); were not reporting on humans; or did not constitute original research.

Articles written in a non-English language were translated to English through direct translation by a native speaker in the authorship team or identified through the authors' networks.

##### 2.1.1.1 Article selection

All members of the research team contributed to article selection. Articles for full text screening were downloaded and imported into Covidence. A minimum of two members of the research team independently screened each title and abstract. Disagreements were discussed until consensus was reached, and a third reviewer was invited to adjudicate when required. The citations and reason(s) for article exclusion from the full text screening stage were recorded. A PRISMA flow diagram was generated to present the search results and screening of the articles for exclusion and inclusion results.

### 2.2 Data extraction and appraisal

Data from the included studies were extracted by one reviewer and cross-checked by a second reviewer. Any disagreements were resolved by two other authors. Data were extracted for the following domains: study reference, year, location, methodology, sample size and characteristics of sample, preconception risk factor, measurement instrument, primary and secondary outcomes measured, duration of study, and key findings. Included studies were critically appraised using the Newcastle-Ottawa Quality Assessment Scale for Cohort studies.

### 2.3 Data synthesis and analysis

**Figure 1:**
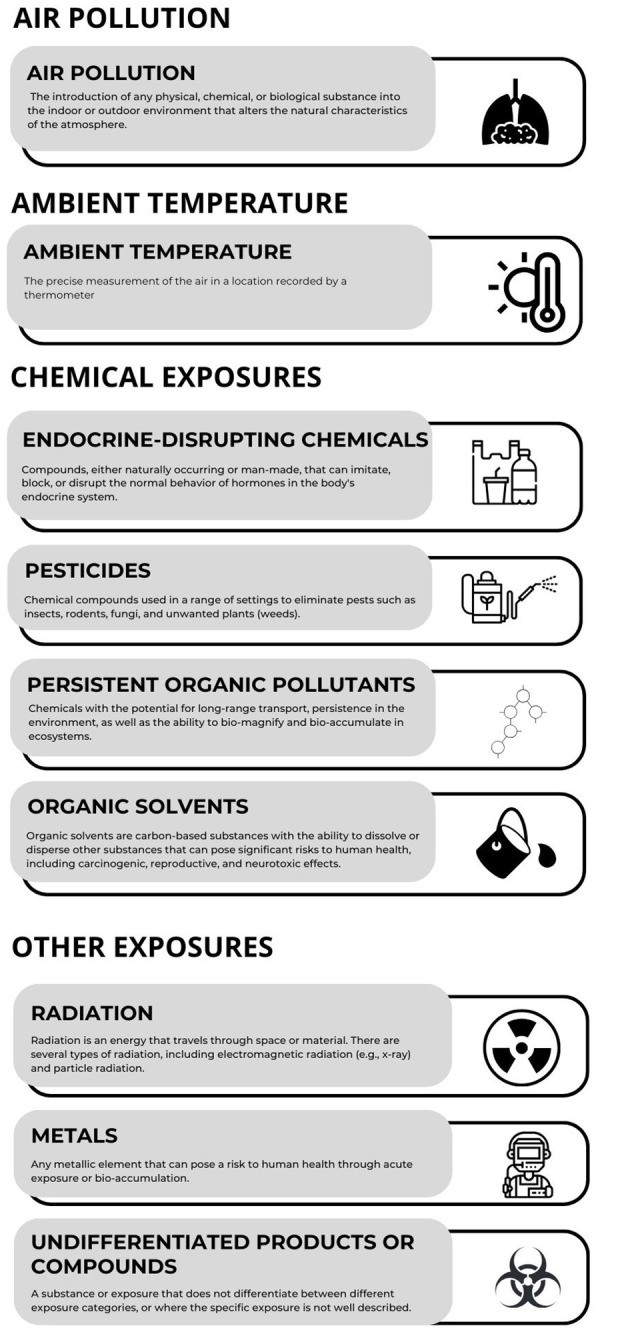
Environmental exposure category definitions employed for data synthesis.

The characteristics of the included studies were presented in tables, summarizing the exposure, main outcomes measured, and study characteristics. Study findings were grouped into categories of environmental exposures and described narratively. A definition of each exposure category is provided in Figure 1. Meta-analysis was not possible due to the heterogeneity of the included studies.

## 3 Results

### 3.1 Study characteristics

**Figure 2:**
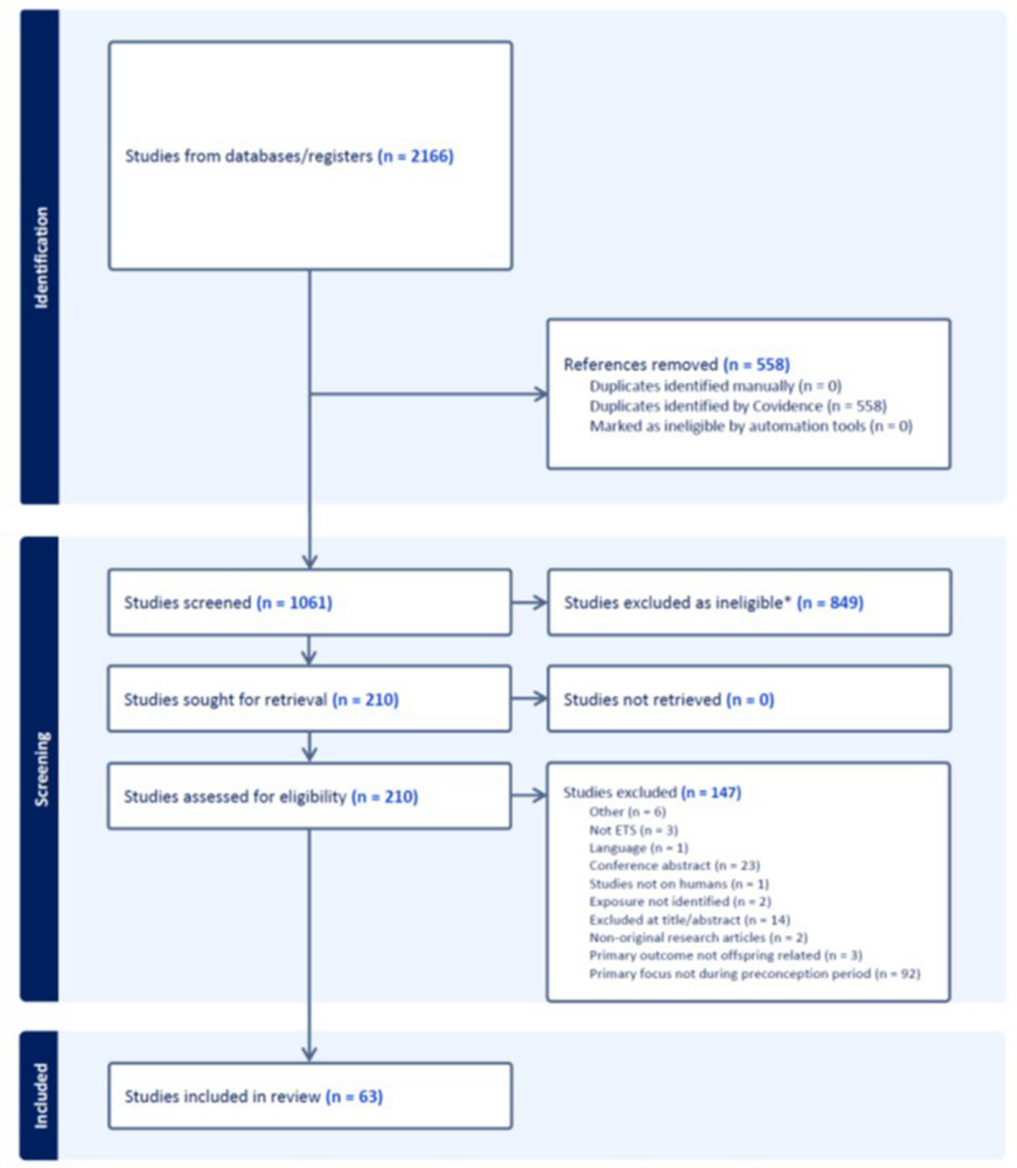
PRISMA diagram depicting the flow of information and manuscript selection. *Eligibility criteria: exposure to any environmental hazard and measured neonatal outcomes or child health outcomes in later life. Environmental exposures were defined as hazards transmitted through environmental media and not managed by health modalities or behavior change (e.g., maternal smoking). The neonatal (>22 weeks completed gestation) outcomes of interest were stillbirth, birthweight, birth length, head circumference, gestational age, and preterm birth. Articles excluded if the environmental exposure could not be clearly attributed to the preconception period. This included studies that examined the peri-conceptual period (3-12 months prior to end first trimster) without differentiating data from before conception. Studies were excluded if the outcomes were not related to birth, neonatal outcomes, or child health (i.e., fertility or maternal outcomes); were not reporting on humans; or did not constitute original research.

**Figure 3:**
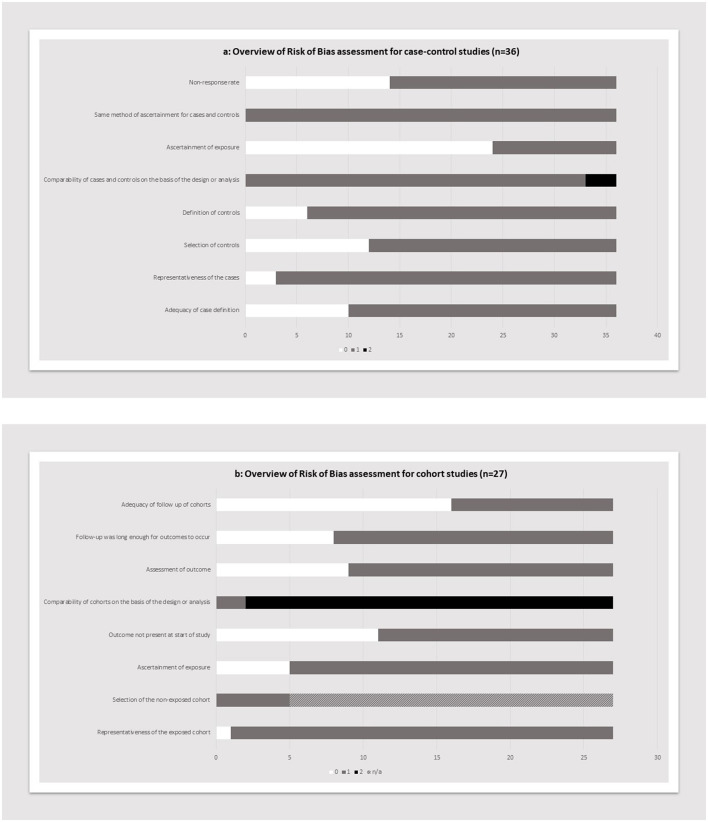
Overview of risk of bias assessment using Newcastle-Ottawa Quality Assessment Scale, for case-control studies **(a)** and cohort studies **(b)**.

The review identified 63 publications that met the inclusion criteria, published between 1974 and 2022 (see Figure 2). The risk of bias scores for the included studies ranged from a minimum of 3 to a maximum of 8 (Median = 6) (see Supplementary Table 2). For the case-control studies, the most common areas not reported were the process of ascertaining exposure, and the non-response rate (Figure 3a). The two most common domains missing for the included cohort studies (Figure 3b) was the adequacy of follow up of cohorts, and the confirmation that the outcome was not present at the start of the study. The studies covered a diverse range of environmental exposure types and were categorized into air pollution (*n* = 21), ambient temperature (*n* = 3), chemicals (*n* = 26), and other general exposure categories (*n* = 24).

### 3.2 Air pollution

The review identified 21 publications investigating maternal and paternal preconception ambient exposures on the outcomes of interest (see Supplementary Table 3). Most studies were conducted in the United States (*n* = 11) and China (*n* = 6) and included data from 2000 to 2020. The preconception exposure periods were commonly 3 months (*n* = 17), with the longest preconception period 18 years (17). All studies investigated outcomes associated with maternal exposures while four studies also investigated paternal exposures (17–20). All studies in this category reported on air pollution exposures such as PM_2.5_, PM_10_, NO_X_, NO_2_, O_3_, SO_2_, CO, dusts, BC, and diesel and petrol exhaust (see Table 1). The most common exposure assessment method was acquiring data from local, regional or state air quality monitoring stations (*n* = 9), followed by using modified Community Multiscale Air Quality (CMAQ) models developed by the U.S. Environmental Protection Agency (EPA) (*n* = 4), self-reported questionnaire (*n* = 2), interviewer administered questionnaire (*n* = 1), geospatial interpolation method (*n* = 2), land-use regression models (*n* = 2), and meteorological models (*n* = 1).

**Table 1:** Outcomes associated with air pollution (*n* = 21) and ambient temperature (*n* = 3).

**Outcome category**	**Study**	**Risk of bias score**	**Preconception exposure period (intensity)**	**Exposure population**	**Environmental exposure**	**Outcomes interested**	**Outcome**
**Air pollution**							
Congenital malformations	Liu et al. (23)	8	3 months (non-occupational)	Maternal	PM_10_	Oral cleft	Increased risk of oral cleft OR 1.04 (95% CI 1.01, 1.07) per 10 μg/m^3^ increment
	Huang et al. (21)	7	3 months (non-occupational)	Maternal	PM_10_, PM_2.5_, NO_x_, NO_2_, O_3_	Hyospadia	NS
	Jiang et al. (22)	6	3 months (non-occupational)	Maternal	SO_2_	Polydactyly	Increased risk of polydactyly OR 3.76 (95% CI 2.61, 5.42)
					SO_2_	Syndactyly	Increased risk of syndactyly OR 3.72 (95% CI 2.05, 6.75)
	Ren et al. (24)	6	1 to 2 months (non-occupational)	Maternal	PM_2.5_	Congenital malformation	NS
	Zhang et al. (26)	5	3 months (non-occupational)	Maternal	PM_10_	Polydactyly	Increased risk of polydactyly OR (range) 1.04–1.95 (95% CI 1.00, 2.45)
					PM_10_	Syndactyly	Increased risk of syndactyly OR (range) 1.08–2.86 (95% CI 1.02, 4.13)
	Yao et al. (25)	5	3 months (non-occupational)	Maternal	PM_10_, NO_2_	Unspecified congenital malformation	NS
					SO_2_	Unspecified congenital malformation	Increased risk of congenital malformation OR 1.20 (95% CI 1.09, 1.29)
	Zhu et al. (27)	5	3 months (non-occupational)	Maternal	CO	Isolated cleft palate	Increased risk of cleft palate OR 2.24 (95% CI 1.21, 4.16)
						Isolated cleft lip	NS
					SO_2_	Isolated cleft palate	NS
						Isolated cleft lip	Increased risk of cleft lip OR 1.93 (95% CI 1.16, 3.21)
					PM_10_	Isolated cleft palate	Increased risk of cleft palate OR 1.72 (95% CI 1.12, 2.66)
						Isolated cleft lip	NS
					NOx, O_3_, PM_2.5_	Isolated cleft palate	NS
						Isolated cleft lip	NS
Adverse birth events	Han et al. (31)	8	3 months (non-occupational)	Maternal	PM_2.5_ level	Premature rupture of membranes	NS
	Mendola et al. (33)	7	3 months (non-occupational)	Maternal	NO_x_	Pre-term birth	Increased risk of pre-term birth RR 1.28 (95% CI 1.13, 1.45)
					CO	Pre-term birth	Increased risk of pre-term birth RR 1.12 (95% CI 1.01, 1.23)
					PM_2.5_, PM_10_, O_3_, SO_2_	Pre-term birth	NS
	Qu et al. (19)	7	Up to 6 months (non-occupational)	Maternal and paternal	Passive cigarette smoking	Stillbirth	*Maternal exposure* Increased stillbirth OR: 1.90 (1.06, 3.39)	NS
	Nobles et al. (34)	7	3 months (non-occupational)	Maternal	O_3_	Small for gestational age	Decreased risk of small for gestational age RR 0.96 (95% CI 0.93, 0.98) per each interquartile range increase
						Fetal growth restriction	Decreased risk of fetal growth restriction RR 0.88 (95% CI 0.83, 0.94)
					NO_x_	Small for gestational age	Increased risk of small for gestational age RR 1.23 (95% CI 1.10, 1.38) per each interquartile range increase
						Fetal growth restriction	Increased risk of fetal growth restriction RR 1.23 (95% CI 1.10, 1.38) per each interquartile range increase
					S0_2_	Small for gestational age	NS
						Fetal growth restriction	Increased risk of fetal growth restriction RR 1.15 (95% CI 1.09, 1.23)
					N0_2_	Small for gestational age	NS
						Fetal growth restriction	Increased risk of fetal growth restriction RR 1.19 (95% CI 1.08, 1.31)
					CO	Small for gestational age	NS
						Fetal growth restriction	Increased risk of fetal growth restriction RR 1.15 (95% CI 1.05, 1.26)
					PM_2.5_	Small for gestational age	Increased risk of fetal growth restriction RR 1.07 (95% CI 1.02, 1.13)
						Fetal growth restriction	NS
					PM_10_	Small for gestational age	NS
						Fetal growth restriction	Increased risk of fetal growth restriction RR 1.13 (95% CI 1.06, 1.21)
	Mekonnen et al. (32)	6	3 months (non-occupational)	Maternal	PM_2.5_	Pre-term birth	NS
					O_3_	Pre-term birth	Increased risk of pre-term birth OR 1.08 (95% CI 1.06, 1.10) for every 1-unit (ppb) increase in O_3_
	Seeni et al. (35)	5	3 months (non-occupational)	Maternal	CO	Transient tachypnea of the newborn	NS
						Asphyxia	NS
						Respiratory distress syndrome	Reduced risk of respiratory distress syndrome RR 0.73 (95% CI 0.68, 0.78)
					NO_x_	Transient tachypnea of the newborn	NS
						Asphyxia	NS
						Respiratory distress syndrome	Increased risk of respiratory distress syndrome RR 1.39 (95% CI 1.25 - 1.54)
					O_3_	Transient tachypnea of the newborn	NS
						Asphyxia	Increased risk of asphyxia RR 1.76 (95% CI 1.25, 2.48)
						Respiratory distress syndrome	Increased risk of respiratory distress syndrome RR 1.09 (95% CI 1.01, 1.18)
					PM_10_	Transient tachypnea of the newborn	Increased risk of transient tachypnea RR 1.10 (95% CI 1.04, 1.17)
						Asphyxia	Reduced risk of asphyxia RR 0.77 (95% CI 0.67, 0.89)
						Respiratory distress syndrome	NS
					PM_2.5_	Transient tachypnea of the newborn	NS
						Asphyxia	NS
						Respiratory distress syndrome	Reduced risk of respiratory distress syndrome RR 0.83 (95% CI 0.76, 0.91)
					SO_2_	Transient tachypnea of the newborn	Reduced risk of transient tachypnea RR 0.84 (95% CI 0.79, 0.90)
						Asphyxia	Reduced risk of asphyxia RR 0.47 (95% CI 0.30, 0.74)
						Respiratory distress syndrome	Reduced risk of respiratory distress syndrome RR 0.76 (95% CI 0.71, 0.82)
	Ha et al. (28–30)	5	3 months (non-occupational)	Maternal	PM constituents: elemental carbon	Stillbirth	NS
						Small for gestational age	Increased risk of small for gestational age RR 1.04 (95% CI 1.01, 1.07)
						Term low birth weight (>37 weeks)	NS
						Early pre-term birth (< 34 weeks)	NS
						Late pre-term birth (34–36 weeks)	NS
						Early term birth (37–38 weeks)	NS
					PM constituents: dust particles	Stillbirth	NS
						Small for gestational age	NS
						Term low birth weight (>37 weeks)	Increased risk of term low birth weight RR 1.10 (95% CI 1.03, 1.17)
						Early pre-term birth (< 34 weeks)	NS
						Late pre-term birth (34–36 weeks)	NS
						Early term birth (37–38 weeks)	NS
					CO, NO_x_, O_3_, PM_2.5_, PM_10_, SO_2_, Other PM constituents (organic compounds, ammonium ions, sulfate particles, nitrate particles)	Stillbirth	NS
						Small for gestational age	NS
						Early pre-term birth (< 34 weeks)	NS
						Late pre-term birth (34–36 weeks)	NS
						Early term birth (37-38 weeks)	NS
Childhood illness	Mao et al. (38)	8	3 months (non-occupational)	Maternal	PM_2.5_	Childhood overweight and obesity	Increased risk of childhood overweight and obesity Q1 v Q3: RR 1.3 (95% CI 1.1, 1.5) Q1 v Q4: RR 1.1 (95% CI 1.0, 1.2)
	Kalkbrenner et al. (37)	8	2.6 months (non-occupational)	Maternal	PM_10_	Autism spectrum disorder	NS
	Talbott et al. (39)	8	3 months (non-occupational)	Maternal	PM_2.5_	Autism spectrum disorder	NS
	Jo et al. (36)	7	3 months (non-occupational)	Maternal	PM_2.5_	Autism spectrum disorder	Increased risk of autism spectrum disorder OR 1.11 (95% CI 1.03, 1.20)
					O_3_, PM_10_ and NO_2_	Autism spectrum disorder	NS
	Schüz et al. (20)	6	12 months (Occupational)	Maternal and Paternal	Industrial dusts	Acute lymphocytic leukemia	*Maternal exposure* NS	*Paternal exposure* Increased risk of acute lymphocytic leukemia OR 1.3 (95% CI 1.0-1.6)
	Miligi et al. (18)	6	12 months (occupational)	Maternal and Paternal	Diesel exhaust	Leukemia	*Maternal exposure* OR: NS	*Paternal exposure* Increased risk of leukemia OR: 1.5 (1.2, 2.0)
						Acute lymphocytic leukemia	*Maternal exposure* OR: NS	*Paternal exposure* Increased risk of acute lymphocytic leukemia OR: 1.6 (1.2, 2.1)
					Petrol exhaust	Leukemia	*Maternal exposure* OR: NS	*Paternal exposure* Increased risk of leukemia OR: 1.6 (1.1, 2.4)
						Acute lymphocytic leukemia	*Maternal exposure* OR: NS	*Paternal exposure* Increased risk of acute lymphocytic leukemia OR: 1.7 (1.1, 2.5)
	Kuiper et al. (17)	4	Birth to 18 years (non-occupational)	Maternal	PM_2.5_	Asthma	Increased risk of asthma from maternal exposure Medium vs. low exposure: OR 2.23 (95% CI 1.32, 3.78) High vs. low exposure: NS
						Hayfever	NS
					PM_10_	Asthma	Increased risk of asthma from maternal exposure Medium vs. low exposure: OR 2.27 (95% CI 1.36, 3.80) High vs. low exposure: NS
						Hayfever	Increased risk of hayfever from maternal exposure Medium vs. low exposure: NS High vs. low exposure: OR 2.66 (95% CI 1.19, 5.91)
					BC. O_3_, No_2_, NDVI	Asthma	NS
						Hayfever	NS
				Paternal	BC	Asthma	Increased risk of asthma from paternal exposure Medium vs. low exposure: NS High vs. low exposure: OR 0.31 (95% CI 0.11, 0.87)
						Hayfever	NS
					O_3_	Asthma	NS
						Hayfever	Increased risk of hayfever from paternal exposure Medium vs. low exposure: OR 4.15 (95% CI 1.28, 13.50) High vs. low exposure: NS
					NO_2_, PM_2.5_, PM_10_, NDVI	Asthma	NS
						Hayfever	NS
**Ambient temperature**							
Ha et al. (28–30)		5	3 months (non-occupational)	Maternal	Ambient temperature, cold (< 10th percentile)	Stillbirth	NS
						Small for gestational age	NS
						Early pre-term birth (< 34 weeks)	NS
						Late pre-term birth (34–36 weeks)	Decreased risk of late pre-term birth OR 0.92 (95% CI 0.86, 0.98)
						Early term birth (37–38 weeks)	Decreased risk of early term birth OR 0.95 (95% CI 0.92, 0.97)
					Ambient temperature, hot (>90th percentile)	Stillbirth	NS
						Small for gestational age	NS
						Early pre-term birth (< 34 weeks)	NS
						Late pre-term birth (34–36 weeks)	Increased risk of late pre-term birth OR 1.09 (95% CI 1.02, 1.16)
						Early term birth (37–38 weeks)	Increased risk of early term birth OR 1.03 (95% CI 1.00, 1.05)

The studies investigating exposure to air pollution measured congenital malformations (*n* = 7) (21–27), adverse birth events (*n* = 7) (19, 28–35) and childhood illnesses (*n* = 7) (17, 18, 20, 36–39) as outcomes (see Table 1). While the components of air pollution varied across studies, some associations were found for a range of neonatal and child health outcomes. An increased risk of oral cleft was reported to be associated with maternal exposure to PM_10_ (23, 27), CO (27), and SO_2_ (27). Risk of polydactyly or syndactyly was found to be associated with PM_10_ (26) and SO_2_ (22). Maternal exposure to ozone was associated with an increased likelihood of pre-term birth in one study (OR 1.08 per unit increase) (32) but no relationship was found for another study (33). Exposure to CO (RR 1.12) (33) among mothers was associated with an increased risk of preterm birth while maternal (but not paternal) exposure to passive cigarette smoking increased the likelihood of stillbirth (OR 1.90) (19). Increased risk of fetal growth restriction was reported for maternal exposure to SO_2_ (RR 1.15), NO_x_ (RR 1.23), CO (RR 1.15), and PM_10_ (RR 1.13) (34). Air pollution was found to increase the risk of a range of neonatal respiratory complications such as respiratory distress syndrome (i.e., NO_x_, and O_3_), asphyxia (i.e., O_3_), and transient tachypnoea (PM_10_). However, some air pollutants (PM_10_ and SO_2_) were found to reduce the risk of these same conditions, with the authors hypothesing there may be a compensatory mechanism in play (35).

A small yet diverse range of childhood illnesses correlated with maternal exposure to air pollutants in five studies. These included an increased risk of childhood overweight and obesity in children between 2 and 9 years old whose mother had preconception exposure to PM_2.5_ (38). PM_2.5_ exposure was also linked with increased risk of autism spectrum disorder in one of the studies (36). Paternal occupational exposure to industrial dusts, diesel exhaust or petrol exhaust in the 12 months before conception increased the odds of their child developing leukemia (18).

### 3.3 Ambient temperature

Three papers were identified from secondary analysis of the Air Quality and Reproductive Health cohort study (28–30). This study was cross-sectional with total participants of 223,375 and exposure was assessed by matching hospital referral zones to modeled meterological data (see Supplementary Table 4). Each paper reported on a different outcome (see Table 1). Maternal exposure to high ambient temperature (90th percentile) in the 3 months before conception was associated with increased odds of late pre-term (34–36 weeks) birth (OR 1.09). A similar association was found with early term birth (37–38 weeks) for this same percentile (OR 1.03). Conversely, maternal exposure to cold ambient temperature (< 10th percentile) was associated with decreased risk of late pre-term birth (OR 0.92) and early term birth (OR 0.95).

### 3.4 Chemical exposures

Supplementary Table 5 summarizes the characteristics of the studies that reported outcomes associated with parental exposure to chemicals (*n* = 26). These studies were published between 1989 and 2021 and were from the United States (*n* = 13), Canada (*n* = 2), Mexico (*n* = 2), China (*n* = 2), Italy (*n* = 2), Colombia (*n* = 1), Ethiopia (*n* = 1), Australia (*n* = 1), Denmark (*n* = 1), and a multi-cohort study involving four countries. Both maternal and paternal population exposures were investigated for 14 studies, with the remainder either focusing on maternal (*n* = 8) or paternal (*n* = 3) populations alone. The studies investigated exposure to endocrine-disrupting chemicals (*n* = 4), pesticides (*n* = 10), persistent organic pollutants (*n* = 4), and organic solvents (*n* = 7). Only 8 of these studies measured chemical exposure in biological specimens (serum = 3, urine = 5). The remainer relied on questionnaires/interviews (*n* = 15) or job exposure matrices (*n* = 3) (see Table 2).

**Table 2:** Outcomes associated with exposure to chemicals (*n* = 26).

**Outcome category**	**Study**	**Risk of bias score**	**Preconception exposure period (intensity)**	**Exposure population**	**Environmental exposure**	**Outcomes interested**	**Outcome**	
**Endocrine-disrupting chemicals**								
Adverse birth outcomes	Zhang et al. (40)	7	Not specified (non-occupational)	People undergoing assisted contraception (no time frame provided). Maternal exposure, although model 3 controls for paternal exposure too.	Σ DEHP (di-(2-ethylhexyl) phthalate)	Preterm birth	*Maternal exposure* Increased risk of preterm birth RR: 1.50 (95% CI 1.09, 2.06)	*Paternal exposure* RR: NS
					MEHP (mono(2-ethylhexyl) phthalate)	Pre-term birth	*Maternal exposure* Increased risk of preterm birth RR: 1.51 (95% CI 1.08, 2.13)	*Paternal exposure* RR: NS
					MEHHP (mono(2-ethyl-5-hydroxyhexyl) phthalate)	Pre-term birth	*Maternal exposure* Increased risk of preterm birth RR: 1.45 (95% CI 1.08, 1.95)	*Paternal exposure* RR: NS
					MEOHP (mono(2-ethyl-5-oxohexyl) phthalate)	Pre-term birth	*Maternal exposure* Increased risk of preterm birth RR: 1.48 (95% CI 1.10, 2.00)	*Paternal exposure* RR: NS
					MECPP (mono(2-ethyl-5-carboxypentyl) phthalate)	Pre-term birth	*Maternal exposure* Increased risk of preterm birth RR: 1.49 (95% CI 1.07, 2.07)	*Paternal exposure* RR: NS
					Σ AAPhthalates	Pre-term birth	*Maternal exposure* Increased risk of preterm birth RR: 1.51 (95% CI 1.08, 2.11)	*Paternal exposure* RR: NS
					MBP (mono-n-butyl phthalate) MiBP (mono-isobutyl phthalate) MBzP (monobenzyl phthalate) MCPP (mono(3-carboxypropyl) phthalate) MCNP (monocarboxyisononyl phthalate) MCOP (monocarboxyisooctyl phthalate) MEP (monoethyl phthalate) MHiNCH (cyclohexane-1,2-dicarboxylic acid monohydroxy isononyl ester) MCOCH (cyclohexane-1,2-dicarboxylic acid monocarboxyisooctyl ester)	Pre-term birth	*Maternal exposure* RR: NS	*Paternal exposure* RR: NS
	Mustieles et al. (42)	7	Not specified (non-occupational)	Maternal and paternal	Bisphenol A	Birth weight	*Maternal exposure* Reduced birth weight Model 1: β −119 g (95% CI: −212, −27) Model 2: β: −79 g (95% CI: −153, −5) Model 3: β: NS Model 1 T1 vs. T2: NS T1 vs. T3: β = −157 (95% CI: −300, −13) Model 2 T1 vs. T2: NS T1 vs. T3: β = −130 (95% CI: −245, −15)	*Paternal exposure* Model 1: NS Model 2: NS Model 3: NS Model 1 T1 vs. T2: NS T1 vs. t3: NS Model 2 T1 vs. T2: NS T1 vs. T3: NS
						Head circumference	*Maternal exposure* Reduced head circumference Model 1: β −0.72 cm (95% CI: −1.3, −0.16) Model 2: β −0.63 cm (95% CI: −1.2, −0.09)	*Paternal exposure* Model 1 β: NS Model 2 β: NS
					Bisphenol S	Birth weight	*Maternal exposure* Increased birth weight Model 1: NS Model 2: NS Model 3: β = 144 g (95% CI: 2, 286)	*Paternal exposure* N/A
						Head circumference	*Maternal exposure* NS	*Paternal exposure* N/A
	Smarr et al. (43)	7	Not specified (non-occupational)	Maternal and paternal	*Low molecular phthalate* mMP (monomethyl phthalate)	Birth weight	*Maternal exposure* Q1 vs. Q2: β = −177.6 (−344.9, −10.3) Q1 vs. Q3: NS Q1 vs. Q4: NS	*Paternal exposure* N/A
						Birth size	*Maternal exposure* Q1 vs. Q2: β −1.6 (−2.6, −0.5) Q1 vs. Q3: β −1.5 (−2.8, −0.1) Q1 vs. Q4: NS	*Paternal exposure* N/A
						Gestational age	*Maternal exposure* Q1 vs. Q2: β −5.5 (−10.0, −1.0) Q1 vs. Q3: NS Q1 vs. Q4: NS	*Paternal exposure* N/A
						Ponderal index		
					*Low molecular phthalate* mEP (monoethyl phthalate)	Birth weight	*Maternal exposure* Q1 vs. Q2: NS Q1 vs. Q3: β −200.2 (−386.9, −13.4) Q1 vs. Q4: NS	*Paternal exposure* N/A
						Head circumference	*Maternal exposure* Q1 vs. Q2: NS Q1 vs. Q3: β −1.4 (−2.3, −0.6) Q1 vs. Q4: NS	*Paternal exposure* N/A
						Birth size	*Maternal exposure* Q1 vs. Q2: NS Q1 vs. Q3: NS Q1 vs. Q4: NS	*Paternal exposure* N/A
						Ponderal index		
						Gestational age		
					*DEHP metabolites* mEHP (monoethylhexyl phthalate)	Birth size	*Maternal exposure* Q1 vs. Q2: NS Q1 vs. Q3: NS Q1 vs. Q4: NS	*Paternal exposure* Q1 vs. Q2: β – 191.93 (−381.61, −2.25) Q1 vs. Q3: NS Q1 vs. Q4: NS
						Gestational age	*Maternal exposure* Q1 vs. Q2: NS Q1 vs. Q3: β 5.7 (0.5, 10.8) Q1 vs. Q4: NS	*Paternal exposure* Q1 vs. Q2: NS Q1 vs. Q3: NS Q1 vs. Q4: NS
						Birth weight	*Maternal exposure* Q1 vs. Q2: NS Q1 vs. Q3: NS Q1 vs. Q4: NS	*Paternal exposure* Q1 vs. Q2: NS Q1 vs. Q3: NS Q1 vs. Q4: NS
						Head circumference		
						Ponderal index		
					*DEHP metabolites* mEOHP (mono-(2-ethyl-5-oxohexyl)phthalate)	Head circumference	*Maternal exposure* Q1 vs. Q2: NS Q1 vs. Q3: β −1.3 (−2.2, −0.4) Q1 vs. Q4: NS	*Paternal exposure* Q1 vs. Q2: NS Q1 vs. Q3: NS Q1 vs. Q4: NS
						Gestational age	*Maternal exposure* Q1 vs. Q2: NS Q1 vs. Q3: NS Q1 vs. Q4: NS	*Paternal exposure* Q1 vs. Q2: β 7.23 (2.36, 12.1) Q1 vs. Q3: β 5.13 (0.02, 10.25) Q1 vs. Q4: β NS
						Birth size		
						Ponderal index		
					*DEHP metabolites* mECPP (mono(5-carboxy-2-ethylpentyl)phthalate)	Head circumference	*Maternal exposure* Q1 vs. Q2: β −0.9 (−1.8, −0.1) Q1 vs. Q3: NS Q1 vs. Q4: NS	*Paternal exposure* Q1 vs. Q2: NS Q1 vs. Q3: NS Q1 vs. Q4: NS
						Gestational age	*Maternal exposure* Q1 vs. Q2: NS Q1 vs. Q3: NS Q1 vs. Q4: NS	*Paternal exposure* Q1 vs. Q2: β 7.33 (1.95, 12.71) Q1 vs. Q3: β NS Q1 vs. Q4: β 6.38 (0.26, 12.5)
						Birth weight	*Maternal exposure* Q1 vs. Q2: NS Q1 vs. Q3: NS Q1 vs. Q4: NS	*Paternal exposure* Q1 vs. Q2: NS Q1 vs. Q3: NS Q1 vs. Q4: NS
						Birth size		
						Ponderal index		
					*DEHP metabolites* mCMHP (mono-[(2-carboxymethyl)hexyl]phthalate)	Birth weight	*Maternal exposure* Q1 vs. Q2: β −201.7 (−372.7, −30.7) Q1 vs. Q3: NS Q1 vs. Q4: NS	*Paternal exposure* N/A
						Birth size	*Maternal exposure* Q1 vs. Q2: NS Q1 vs. Q3: NS Q1 vs. Q4: NS	*Paternal exposure* N/A
						Head circumference		
						Ponderal index		
						Gestational age		
					*High molecular weight* mOP (mono-n-octylphthalate)	Birth weight	*Maternal exposure* Q1 vs. Q2: β −215.4 (−387.1, −43.7) Q1 vs. Q3: NS	*Paternal exposure* Q1 vs. Q2: NS Q1 vs. Q3: NS Q1 vs. Q4: NS
						Birth size	*Maternal exposure* Q1 vs. Q2: NS Q1 vs. Q3: NS	*Paternal exposure* Q1 vs. Q2: β −1.22 (−2.33, −0.12) Q1 vs. Q3: NS Q1 vs. Q4: NS
						Gestational age	*Maternal exposure* Q1 vs. Q2: β −5.2 (−9.9, −0.4) Q1 vs. Q3: NS	*Paternal exposure* Q1 vs. Q2: NS Q1 vs. Q3: NS Q1 vs. Q4: NS
						Ponderal index		
					*High molecular weight* mCHP (monocyclohexylphthalate)	Birth weight	*Maternal exposure* N/A	*Paternal exposure* Q1 vs. Q2: NS Q1 vs. Q3: NS Q1 vs. Q4: β 224.45 (33.94, 414.96)
						Gestational age	*Maternal exposure* N/A	*Paternal exposure* Q1 vs. Q2: NS Q1 vs. Q3: NS Q1 vs. Q4: β 7.01 (2.16, 11.86)
						Birth size	*Maternal exposure* N/A	*Paternal exposure* Q1 vs. Q2: NS Q1 vs. Q3: NS Q1 vs. Q4: NS
						Head circumference		
						Ponderal index		
					BPA (bisphenol A)	Birth size	*Maternal exposure* N/A	*Paternal exposure* Q1 vs. Q2: NS Q1 vs. Q3: NS Q1 vs. Q4: β 1.35 (0.25, 2.45)
						Birth weight	*Maternal exposure* N/A	*Paternal exposure* Q1 vs. Q2: NS Q1 vs. Q3: NS Q1 vs. Q4: NS
						Head circumference		
						Ponderal index		
						Gestational age		
	Zhang et al. (41)	5	Not specified (non-occupational)	Maternal and paternal	DEHP-BPA factor	Pre-term birth, gestation < 259 days	*Maternal exposure* Increased risk of pre-term birth RR 1.36 (95% CI: 1.00, 1.84)	*Paternal exposure* NS
					Paraben factor, high molecular weight phthalate factor, low molecular weight phthalate factor	Pre-term birth, gestation < 259 days	*Paternal exposure* NS	*Paternal exposure* NS
**Pesticides**								
Congenital malformations	Lacasaña et al. (44)	7	3 months (occupational)	Maternal and paternal	Occupational exposure to agricultural work (inclusive of but not limited to pesticides)	Anencephaly	*Maternal exposure* NS	*Paternal exposure* NS
	Qu et al. (19)	6	Within 6 months (not specified)	Maternal and paternal	Pesticides (unspecified)	Congenital malformations (unspecified)	NS	
	Addissie et al. (45)	4	3 months (occupational and non-occupational)	Maternal	Any personal insect repellents (contained DEET, did not contain DEET, with and without DEET), weed killers, pesticides for pets, household pest control products, occupational exposures to pesticides and herbicides	Holoprosencephaly	NS	
	Ly et al. (46)	4	12-18 months (occupational)	Paternal	Agricultural chemical	Orofacial cleft	NS	
					Agent Orange	Orofacial cleft	NS	
	Weselak et al. (48)	3	3 months (occupational)	Maternal or paternal (undifferentiated)	Herbicides	Unspecified birth defects	*Reported farm chemical use* Decreased risk of birth defects in female offspring All offspring: NS Male offspring: NS Female offspring: OR 0.36 (95% CI 0.14, 0.93)	*Direct chemical activity* Decreased risk of birth defects in offspring All offspring: OR 0.53 (95%CI 0.29, 0.96)
					Fungicides	Unspecified birth defects	*Reported farm chemical use* All offspring: NS Male offspring: NS Female offspring: NS	*Direct chemical activity* All offspring: NS
					Insecticides	Unspecified birth defects	*Reported farm chemical use* All offspring: NS Male offspring: NS Female offspring: NS	*Direct chemical activity* All offspring: NS
					Other pesticides	Unspecified birth defects	*Reported farm chemical use* All offspring: NS Male offspring: NS	
					Phenoxy herbicides	Unspecified birth defects	*Reported farm chemical use* All offspring: NS Male offspring: NS	*Direct chemical activity* Increased risk of birth defects in offspring All offspring: OR 0.42 (0.18, 0.94)
					Organophosphates	Unspecified birth defects	*Reported farm chemical use* All offspring: NS Male offspring: NS	*Direct chemical activity* All offspring: NS
					Thiocarbamates	Unspecified birth defects	*Reported farm chemical use* All offspring: NS Male offspring: NS	*Direct chemical activity* All offspring: NS
					Carbaryl	Unspecified birth defects	*Reported farm chemical use* All offspring: NS Male offspring: NS	
					2,4-D	Unspecified birth defects	*Reported farm chemical use* All offspring: NS Male offspring: NS	*Direct chemical activity* All offspring: NS
					Dicamba	Unspecified birth defects	*Reported farm chemical use* Increased risk of birth defects in male offspring All offspring: NS Male offspring: OR 2.42 (95% CI 1.06, 5.53)	
					Fungicides/Insecticides	Unspecified birth defects	*Reported farm chemical use* All offspring: NS Male offspring: NS	
					Herbicides/Insecticides	Unspecified birth defects	*Reported farm chemical use* All offspring: NS Male offspring: NS	
					Herbicide/Fungicides	Unspecified birth defects	*Reported farm chemical use* All offspring: NS Male offspring: NS	
					Cyanazine	Unspecified birth defects	*Reported farm chemical use* Increased risk of birth defects in male offspring Male offspring: OR 4.99 (95% CI 1.63, 15.27)	*Direct chemical activity* All offspring: NS
Adverse birth outcomes	Hu et al. (98)	8	Not specified (non-occupational)	Maternal	Chemical activity	Unspecified birth defects	*Direct chemical activity* All offspring: NS	
						Clinical pregnancy	Q1 vs. Q2: NS Q1 vs. Q3: RR 0.78 (95% CI 0.65, 0.93) Q1 vs. Q4: RR 0.76 (95% CI: 0.62, 0.92) Reduced pregnancy rate	
						Live birth	Q1 vs. Q2: NS Q1 vs. Q3: RR 0.81 (95% CI 0.68, 0.97) Q1 vs. Q4: RR 0.79 (95% CI 0.66, 0.96) Reduce live birth rate	
					Σ4DAP (diakylphosphate – sum of DMP, DMTP, DEP)	Successful implantation	Q1 vs. Q2: NS Q1 vs. Q3: NS Q1 vs. Q4: RR 0.77 (95% CI 0.60, 0.99) Reduced implantation rate	
						Clinical pregnancy	Q1 vs. Q2: NS Q1 vs. Q3: NS Q1 vs. Q4: RR 0.80 (95% CI 0.65, 0.99) Reduced pregnancy rate	
						Live birth	Q1 vs. Q2: NS Q1 vs. Q3: NS Q1 vs. Q4: NS	
					DMP (dimethylphosphate) DMTP (dimethylthiophosphate) DETP (diethylthiophosphate)	Successful implantation	Q1 vs. Q2: NS Q1 vs. Q3: NS Q1 vs. Q4: NS	
						Clinical pregnancy		
						Live birth		
	Chiu et al. (50)	7	Not specified (non-occupational)	Maternal	Total pesticide residue on fresh produce (servings/day)	Clinical pregnancy	Reduced incidence of clinical pregnancy Q1: OR 0.63 (95% CI 0.51, 0.74) Q2: OR 0.60 (95% CI 0.49, 0.69) Q3: OR 0.65 (95% CI 0.55, 0.73) Q4: OR 0.56 (95% CI 0.41, 0.69)	
						Live birth	Reduced incidence of live birth Q1: OR 0.51 (95% CI 0.39, 0.63) Q2: OR 0.51 (95% CI 0.41, 0.61) Q3: OR 0.60 (95% CI 0.50, 0.69) Q4: OR 0.46 (95% CI 0.32, 0.61)	
					High pesticide residues on fresh produce (servings/day)	Clinical pregnancy	Reduced incidence of clinical pregnancy Q1: OR 0.67 (95% CI 0.55, 0.77) Q2: OR 0.70 (95% CI 0.60, 0.78) Q3: OR 0.58 (95% CI 0.48, 0.67) Q4: OR 0.49 (95% CI 0.37, 0.62)	
						Live birth	Reduced incidence of live birth Q1: OR 0.65 (95% CI 0.52, 0.76) Q2: OR 0.55 (95% CI 0.45, 0.64) Q3: OR 0.49 (95% CI 0.39, 0.59) Q4: OR 0.39 (95% CI 0.28, 0.52)	
					Low pesticide residues on fresh produce (servings/day)	Clinical pregnancy	Reduced incidence of clinical pregnancy Q1: OR 0.50 (95% CI 0.39, 0.61) Q2: OR 0.65 (95% CI 0.54, 0.74) Q3: OR 0.63 (95% CI 0.53, 0.71) Q4: OR 0.67 (95% CI 0.53, 0.78)	
						Live birth	Reduced incidence of live birth Q1: OR 0.38 (95% CI 0.28, 0.50) Q2: OR 0.56 (95% CI 0.45, 0.66) Q3: OR 0.58 (95% CI 0.48, 0.67) Q4: OR 0.57 (95% CI 0.43, 0.69)	
	Qu et al. (19)	6	Within 6 months (not specified)	Maternal and paternal	Pesticides	Low birth weight	Increased risk of low birth weight OR 7.43 (95% CI 5.27, 10.47)	
						Miscarriage	NS	
						Preterm birth	NS	
						Stillbirth	Increased risk of stillbirth OR 3.28 (95% CI: 2.00, 5.40)	
Childhood illnesses	Greenop et al. (51)	7	12 months (occupational and non-occupational)	Maternal and paternal	Pesticides used for pest control	Childhood Brain Tumors	Increased risk of childhood brain tumors No pest control vs. any pest control: NS No pest control vs. any pest control in this period only: OR 1.90 (95% CI 1.08, 3.36)	
					Pesticides used for any termite control Pesticides used for spiders/insects control	Childhood Brain Tumors	NS	
					Paternal occupational exposure to pesticides	Childhood Brain Tumors	NS	
					Pesticides used for pest control	Low grade gliomas	NS	
						High grade gliomas	NS	
						Germ cell tumors	NS	
						Ependymomas	NS	
						Embryonal tumors	NS	
	Slater et al. (71)	7	1 month (non-occupational)	Maternal	Insecticides Moth control Rodenticides Flea or tick control Herbicides Insect repellants Professional pest exterminations	Acute lymphoblastic leukemia	NS	
						Acute myeloid leukemia	NS	
						Mixed lineage leukemia gene rearrangement present	NS	
						No mixed lineage leukemia gene rearrangement present	NS	
**Persistent organic pollutants**								
Birth outcomes	Bae et al. (52)	6	12 months (non-occupational)	Maternal and paternal	Polychlorinated biphenyl congener 128	Male birth rate	*Maternal exposure* NS	*Paternal exposure* Increased male birth rate RR 1.21 (95% CI 1.03, 1.42)
					Hexachlorobenzene	Male birth rate	*Maternal exposure* NS	*Paternal exposure* Decreased male birth rate RR 0.81 (95% CI: 0.68, 0.96)
					Mirex	Male birth rate	*Maternal exposure* Increased male birth rate RR 1.09 (95% CI 1.05, 1.13)	*Paternal exposure* NS
					p,p′-DDE	Male birth rate	*Maternal exposure* Increased male birth rate RR 1.16 (95% CI 1.02, 1.31)	*Paternal exposure* Increased male birth rate RR 1.28 (95% CI 1.10, 1.49)
					PBDE 99	Male birth rate	*Maternal exposure* Increased male birth rate RR 1.20 (95% CI 1.01, 1.44)	*Paternal exposure* NS
					PBDE 154	Male birth rate	*Maternal exposure* Increased male birth rate RR 1.27 (95% CI 1.04, 1.56)	*Paternal exposure* NS
					PBDE 47	Male birth rate	*Maternal exposure* NS	*Paternal exposure* Increased male birth rate RR 1.29 (95% CI 1.06, 1.56)
					PCB 87	Male birth rate	*Maternal exposure* Increased male birth rate RR 1.11 (95% CI 1.00, 1.23)	*Paternal exposure* NS
					PCB 66	Male birth rate	*Maternal exposure* NS	*Paternal exposure* Increased male birth rate RR 1.06 (95% CI 1.00, 1.11)
					PCB 74	Male birth rate	*Maternal exposure* NS	*Paternal exposure* Increased male birth rate RR 1.07 (95% CI 1.00, 1.13)
					Other personal environmental chemicals –, p,p′-DDT, PBDE 17, PBDE 28, PBDE 47, PBDE 85, PBDE 154, PCB 28, PCB 44, PCB 49, PCB 52, PCB 157	Male birth rate	*Maternal exposure* NS	*Paternal exposure* NS
	Murphy et al. (53)	6	Up to 12 months preconception (non-occupational)	Maternal	Antiestrogenic polychlorinated biphenyl (PCBs)	Birth weight	Medium exposure: NS High exposure: β −470.8 g (−890.2, −51.3)	
					Total polychlorinated biphenyl (PCB) Estrogenic PCBs Other PCBs		NS	
	Robledo et al., (54)	6	Not specified (non-occupational)	Maternal & paternal	o,p′-DDT (dichlorodiphenyltrichloroethane)	Birth weight (g), by infant sex	*Maternal exposure* Lower birth weight for female offspring Female: β −195.39 (95% CI −351.25, −39.52) Male: NS	*Paternal exposure* Lower birth weight for male and female offspring Female: β −49.28 (95% CI −153.83, 55.27) Male: β 4.32 (95% CI −86.15, 94.79)
						Head circumference (cm), by infant sex	*Maternal exposure* Lower head circumference for female offspring Female: β −0.78 (05% CI −1.48, −0.09) Male: NS	*Paternal exposure* Female: NS Male: NS
					PBDE-28 (2,4,4′-tribromodiphenyl ether)	Birth weight (g), by infant sex	*Maternal exposure* Lower birth weight for female offspring Female: β −151.33 (95% CI −298.56, −4.10) Male: NS	*Paternal exposure* Female: NS Male: NS
						Body length (cm), by infant sex	*Maternal exposure* Lower body length for female offspring Female: β −1.14 (95% CI −2.00, −0.28) Male: NS	*Paternal exposure* Female: NS Male: NS
					PBDE-66 (2,3′,4,4′-tetrabromodiphenyl ether)	Birth weight (g), by infant sex	*Maternal exposure* Greater birth weight for male offspring Female: NS Male: β 125.04 (95% CI 18.16, 231.92)	*Paternal exposure* Female: NS Male: NS
						Head circumference (cm), by infant sex	*Maternal exposure* Larger head circumference for male offspring Female: NS Male: β 0.60 (95% CI 0.02, 1.18)	*Paternal exposure* Female: NS Male: NS
					PBDE-85 (2,2′,3,4,4′-pentabromodiphenyl ether)	Head circumference (cm), by infant sex	*Maternal exposure* Larger head circumference for male offspring Female: NS Male: β 1.04 (95% CI 0.04, 2.03)	*Paternal exposure* Female: NS Male: NS
					PBDE-99 (2,2′,4,4′,5-pentabromodiphenyl ether)	Birth weight (g), by infant sex	*Maternal exposure* Higher birth weight for male offspring Female: NS Male: β 133.39 (95% CI 9.12, 257.37)	*Paternal exposure* Female: NS Male: NS
						Body length (cm), by infant sex	*Maternal exposure* Longer body length for male offspring Female: NS Male: β 0.76 (95% CI 0.04, 1.48)	*Paternal exposure* Female: NS Male: NS
						Head circumference (cm), by infant sex	*Maternal exposure* Greater head circumference for male offspring Female: NS Male: β 0.91 (95% CI 0.23, 1.60)	*Paternal exposure* Female: NS Male: NS
					PCB-128 (2,2′,3,3′,4,4′-Hexachlorobiphenyl)	Head circumference (cm), by infant sex	*Maternal exposure* Lower head circumference for male offspring Female: NS Male: β −0.86 (95% CI −1.45, −0.10)	*Paternal exposure* Lower head circumference for male offspring Female: NS Male: β −0.66 (95% CI −1.31, −0.01)
					PCB-138 (2,2′,3,4,4′,5′-Hexachlorobiphenyl)	Birth weight (g), by infant sex	*Maternal exposure* Lower birth weight for male offspring Female: NS Male: β −149.6 (95% CI −285.16, −14.06)	*Paternal exposure* Female: NS Male: NS
						Head circumference (cm), by infant sex	*Maternal exposure* Lower head circumference for female and male offspring Female: β −0.65 (95% CI −1.25, −0.05) Male: β −0.67 (95% CI −1.67, −0.06)	*Paternal exposure* Female: NS Male: NS
						Ponderal Index (g/cm^3^), by infant sex	*Maternal exposure* Lower ponderal index for female and male offspring Female: β −0.10 (95% CI −0.20, −0.01) Male: β −0.13 (95% CI −0.23, −0.04)	*Paternal exposure* Lower ponderal index for female and male offspring Female: β −0.09 (95% CI −0.18, 0.00) Male: β −0.13 (95% CI −0.24, −0.02)
					PCB-153 (2,2′,4,4′,5,5′-Hexachlorobiphenyl)	Birth weight (g), by infant sex	*Maternal exposure* Lower birth weight for male offspring Female: NS Male: β −169.93 (95% CI −317.32, −22.53)	*Paternal exposure* Female: NS Male: NS
						Head circumference (cm), by infant sex	*Maternal exposure* N/A	*Paternal exposure* Female: NS Male: NS
					PCB-156 (2,3,3′,4,4′,5-Hexachlorobiphenyl)	Ponderal Index (g/cm^3^), by infant sex	*Maternal exposure* Female: NS Male: NS	*Paternal exposure* Lower ponderal index for female and male offspring Female: β −0.08 (95% CI −0.16, −0.01) Male: β −0.11 (95% CI −0.20, −0.03)
						Ponderal Index (g/cm3), by infant sex	*Maternal exposure* Female: NS Male: NS	*Paternal exposure* Lower ponderal index for male offspring Female: NS Male: β −0.08 (95% CI −0.16, −0.01)
					PCB-167 (2,3′,4,4′,5,5′-Hexachlorobiphenyl)	Birth weight (g), by infant sex	*Maternal exposure* Lower birth weight for male offspring Female: NS Male: β −129.24 (95% CI −228.16, −30.31)	*Paternal exposure* Lower birth weight for female offspring Female: β −97.49 (95% CI −187.45, −7.54) Male: NS
						Body length (cm), by infant sex	*Maternal exposure* N/A	*Paternal exposure* Lower body length for female offspring Female: β −0.57 (95% CI −1.12, −0.02) Male: β = NS
						Head circumference (cm), by infant sex	*Maternal exposure* Lower head circumference for male offspring Female: β = NS Male: β = −0.47 (95% CI −0.95, 0.00)	*Paternal exposure* Lower head circumference for female offspring Female: β = −0.45 (95% CI −0.86, −0.03) Male: NS
					PCB-170 (2,2′,3,3′,4,4′,5-Heptachlorobiphenyl)	Birth weight (g), by infant sex	*Maternal exposure* Lower birth weight for male offspring Female: NS Male: β −153.69 (95% CI −288.45, −18.92)	*Paternal exposure* Female: NS Male: NS
						Body length (cm), by infant sex	*Maternal exposure* Female: NS Male: NS	*Paternal exposure* N/A
						Ponderal Index (g/cm^3^), by infant sex	*Maternal exposure* Lower ponderal index for female and male offspring	*Paternal exposure* Female: NS Male: NS
					PCB-172 (2,2′,3,3′,4,5,5′-Heptachlorobiphenyl)	Birth weight (g), by infant sex	*Maternal exposure* Female: NS Male: NS	*Paternal exposure* Lower birth weight for male offspring Female: NS Male: β −166.89 (95% CI −311.19, −22.60)
						Ponderal Index (g/cm3), by infant sex	*Maternal exposure* Lower ponderal index for male offspring Female: NS Male: β −0.09 (95% CI −0.17, −0.02)	*Paternal exposure* Female: NS Male: NS
					PCB-195 (2,2′,3,3′,4,4′,5,6-Octachlorobiphenyl)	Birth weight (g), by infant sex	*Maternal exposure* Lower birth weight for male offspring Female: NS Male: β −137.73 (95% CI −259.57, −15.89)	*Paternal exposure* Female: NS Male: NS
					PCB-201 (2,2′,3,3′,4,5′,6,6′-Octachlorobiphenyl)	Head circumference (cm), by infant sex	*Maternal exposure* Larger head circumference for female offspring Female: β 0.51 (95% CI 0.08, 0.93) Male: NS	*Paternal exposure* Female: NS Male: NS
					PCB-206 (2,2′,3,3′,4,4′,5,5′,6-Nonachlorobiphenyl)	Head circumference (cm), by infant sex	*Maternal exposure* Larger head circumference for female offspring Female: β 0.52 (95% CI 0.06, 0.98) Male: NS	*Paternal exposure* Female: NS Male: NS
					PCB-209 (Decachlorobiphenyl)	Birth weight (g), by infant sex	*Maternal exposure* Lower birth weight for male offspring Femal: NS Male: β −98.88 (95% CI −187.14, −10.61)	*Paternal exposure* Female: NS Male: NS
					PFOSA (Perfluorooctanesulfonamide)	Birth weight (g), by infant sex	*Maternal exposure* Lower birth weight for male offspring Female: NS Male: β −104.23 (95% CI −194.16, −14.30)	*Paternal exposure* Female: NS Male: NS
					γ-HCH (gamma-hexachlorocyclohexane)	Body length (cm), by infant sex	*Maternal exposure* Lower body length for female offspring Female: β −0.59 (95% CI −1.14, −0.03) Male: NS	*Paternal exposure* Female: NS Male: NS
						Ponderal Index (g/cm^3^), by infant sex	*Maternal exposure* Greater ponderal index for female offspring Female: β 0.09 (95% CI 0.03, 0.16) Male: NS	*Paternal exposure* Greater ponderal index for female offspring Female: β 0.08 (95% CI 0.02, 0.13) Male: NS
					HCB (hexachlorobenzene)	Head circumference (cm), by infant sex	*Maternal exposure* Larger head circumference for male offspring Female: NS Male: β 0.44 (95% CI 0.01, 0.87)	*Paternal exposure* Female: NS Male: β = NS
					β-HCH (β-Hexachlorocyclohexane)	Head circumference (cm), by infant sex	*Maternal exposure* Lower head circumference for male offspring Female: β −1.47 (95% CI −2.33, −0.61) Male: NS	*Paternal exposure* Female: NS Male: NS
					p,p′-DDE (dichlorodiphenyldichloroethylene)	Ponderal Index (g/cm3), by infant sex	*Maternal exposure* Female: NS Male: NS	*Paternal exposure* Greater ponderal index for female offspring Female: β 0.12 (95% CI 0.02, 0.22) Male: NS
					Et-PFOSA-AcOH (2-(N-Ethyl-perfluorooctane sulfonamide) acetic acid)	Ponderal Index (g/cm3), by infant sex	*Maternal exposure* Lower ponderal index for female offspring Female: β −0.09 (95% CI −0.16, −0.02) Male: NS	*Paternal exposure* Female: NS Male: NS
	Weisskopf et al. (55)	4	Not specified (non-occupational)	Maternal	Fish consumption from contaminated area, number of meals	Birthweight	NS	
**Organic solvents**								
Congenital malformations	Aguilar-Garduno et al. (56)	6	Prior to 3 months before conception (up to 5 years)	Maternal and paternal	Non-specified occupational organic solvents	Anencephaly	*Maternal exposure* NS	*Paternal exposure* NS
Childhood illness	Parodi et al. (61)	7	Not specified (occupational)	Maternal	Any solvent	Neuroblastoma	Increased risk of neuroblastoma OR 2.0 (95% CI 1.0, 4.1)	
					Aromatic hydrocarbons	Neuroblastoma	Increased risk of neuroblastoma OR 9.2 (95% CI 2.4, 34.3)	
					Aliphatic hydrocarbons	Neuroblastoma	Increased risk of neuroblastoma OR 5.7 (95% CI 1.3, 24.9)	
					Derivative oxygenate hydrocarbons	Neuroblastoma	Increased risk of neuroblastoma OR 26 (95% CI 1.1, 6.5)	
					Chlorinated hydrocarbons	Neuroblastoma	NS	
	Castro-Jimenez et al., (58)	6	24 months (occupational), maternal and paternal,	Maternal and Paternal	Aliphatic hydrocarbons	Acute lymphocytic leukemia	*Maternal exposure* Increased risk of acute lymphocytic leukemia OR 6.33 (95% CI 1.41–28.31)	*Paternal exposure* NS	*Both parental exposure* Increased risk of acute lymphocytic leukemia OR 13.47 (95% CI 3.31–54.71)
					Amides	Acute lymphocytic leukemia	*Maternal exposure* NS	*Paternal exposure* NS	*Both parental exposure* NS
					Aromatics	Acute lymphocytic leukemia	*Maternal exposure* Increased risk of acute lymphocytic leukemia OR 6.33 (95% CI 1.41–28.31)	*Paternal exposure* NS	*Both parental exposure* Increased risk of acute lymphocytic leukemia OR 13.47 (95% CI 3.31–54.71)
					1,3-Butadiene	Acute lymphocytic leukemia	*Maternal exposure* NS	*Paternal exposure* Increased risk of acute lymphocytic leukemia OR 4.18 (95% CI 1.47–11.88)	*Both parental exposure* NS
					Trichloroethylene	Acute lymphocytic leukemia	*Maternal exposure* Increased risk of acute lymphocytic leukemia OR 7.41 (95% CI 1.66, 33.07)	*Paternal exposure* Increased risk of acute lymphocytic leukemia OR 2.76 (95% CI 1.09–7.06)	*Both parental exposure* Increased risk of acute lymphocytic leukemia OR 17.56 (95% CI 4.12, 74.81)
					Acrylamide	Acute lymphocytic leukemia	*Maternal exposure* NS	*Paternal exposure* NS	*Both parental exposure* NS
					Benzyl chloride	Acute lymphocytic leukemia	*Maternal exposure* NS	*Paternal exposure* NS	*Both parental exposure* Increased risk of acute lymphocytic leukemia OR 7.66 (95% CI 1.20, 48.69)
					Epichlorohydrin	Acute lymphocytic leukemia	*Maternal exposure* Increased risk of acute lymphocytic leukemia OR 6.59 (95% CI 1.05, 41.32)	*Paternal exposure* NS	*Both parental exposure* Increased risk of acute lymphocytic leukemia OR 11.56 (95% CI 1.67, 79.80)
					Ethylene oxide	Acute lymphocytic leukemia	*Maternal exposure* Increased risk of acute lymphocytic leukemia OR 3.85 (95% CI 1.05, 14.09)	*Paternal exposure* NS	*Both parental exposure* Increased risk of acute lymphocytic leukemia OR 7.98 (95% CI 2.10, 30.28)
					Ortho-Toluidine	Acute lymphocytic leukemia	*Maternal exposure* NS	*Paternal exposure* NS	*Both parental exposure* Increased risk of acute lymphocytic leukemia OR 6.44 (95% CI 1.43, 28.94)
	Heck et al. (59)	6	3 months (occupational)	Paternal	Benzene	Acute lymphoblastic leukemia	Model 1: NS Model 2: NS	
						Astrocytoma	Model 1: NS Model 2: NS	
						Germ cell tumors	Model 1: NS Model 2: NS	
						Acute undifferentiated leukemia/acute leukemia not otherwise specified	Increased risk of acute undifferentiated leukemia Model 1: OR 5.77 (95% CI 1.83, 18.17) Model 2: OR 6.11 (95% CI 1.81, 20.61)	
					Tolouene	Acute lymphoblastic leukemia	Model 1: NS Model 2: NS	
	Miligi et al. (18)	6	12 months (occupational)	Maternal and paternal	Any solvent	Leukemia	*Maternal exposure* NS	*Paternal exposure* NS
						Acute lymphocytic leukemia	*Maternal exposure* NS	*Paternal exposure* NS
						Acute non-lymphoblastic leukemia	*Maternal exposure* NS	*Paternal exposure* NS
					Aromatic hydrocarbons	Leukemia	*Maternal exposure* Increased risk of leukemia OR 3.8 (95% CI 1.6 - 9.2)	*Paternal exposure* NS
						Acute lymphocytic leukemia	*Maternal exposure* Increased risk of acute lymphocytic leukemia OR 3.8 (95% CI 1.5, 9.5)	*Paternal exposure* NS
						Acute non-lymphoblastic leukemia	*Maternal exposure* NS	*Paternal exposure* NS
					Chlorinated hydrocarbons	Leukemia	*Maternal exposure* NS	*Paternal exposure* NS
						Acute lymphocytic leukemia	*Maternal exposure* NS	*Paternal exposure* NS
						Acute non-lymphoblastic leukemia	*Maternal exposure* NS	*Paternal exposure* NS
					Oxygenated derivatives of hydrocarbons	Leukemia	*Maternal exposure* Increased risk of leukemia OR 1.9 (95% CI 1.1, 3.2)	*Paternal exposure* NS
						Acute lymphocytic leukemia	*Maternal exposure* Increased risk of acute lymphocytic leukemia OR 1.8 (95% CI 1.0, 3.1)	*Paternal exposure* NS
						Acute non-lymphoblastic leukemia	*Maternal exposure* NS	*Paternal exposure* NS
					Aliphatic hydrocarbons	Leukemia	*Maternal exposure* Increased risk of leukemia OR 4.3 (95% CI 1.8, 10.4)	*Paternal exposure* NS
						Acute lymphocytic leukemia	*Maternal exposure* Increased risk of acute lymphocytic leukemia OR 4.2 (95% CI 1.7, 10.3)	*Paternal exposure* NS
						Acute non-lymphoblastic leukemia	*Maternal exposure* Increased risk of acute non-lymphoblastic leukemia OR 4.2 (95% CI 1.0, 17.2)	*Paternal exposure* NS
					PAH	Leukemia	*Maternal exposure* NS	*Paternal exposure* Increased risk of leukemia OR 1.3 (95% CI 1.0, 1.8)
						Acute lymphocytic leukemia	*Maternal exposure* NS	*Paternal exposure* Increased risk of acute lymphocytic leukemia OR 1.3 (95% CI 1.0, 1.8)
						Acute lymphoblastic leukemia	*Maternal exposure* NS	*Paternal exposure* Increased risk of acute lymphoblastic leukemia OR 1.5 (95% CI 1.1, 2.1)
	Olshan et al. (60)	4	Any time prior to pregnancy	Paternal	Hydrocarbons (broad, narrow, combusted)	Wilms' tumor	NS	
	Cassidy et al. (57)	3	At the time of conception	Paternal	Hydrocarbons (unspecified)	Prader Willi syndrome	NS	

#### 3.4.1 Endocrine-disrupting chemicals

Only adverse birth outcomes associated with exposure to endocrine-disrupting chemicals were investigated for this category (*n* = 4). Overall maternal exposure to plastic additives, such as phthalates and bisphenols, did not demonstrate a consistent effect for a range of neonatal outcomes, and paternal exposure did not appear to have a relationship with the outcomes studied. Two studies from China found maternal exposure to phenol and phthalate compounds increased the risk of preterm birth (RR 1.36–1.51) but paternal exposure did not (40, 41). Maternal exposure to bisphenol A (BPA) and phthalate compounds were also found to reduce birth weight by between 79 g and 119 g, and reduce head circumference by 0.6 cm to 0.72 cm (42) although this finding was not replicated in other work (43). Interestingly, bisphenol S (BPS) was associated with birthweight in the opposite direction, with higher levels of BPS associated with increased birth weight (by 144 g). Preconception phthalate compound exposure was also investigated by this second study and there were no consistent trends associated with either maternal or paternal exposure for any of the birth outcomes measured.

#### 3.4.2 Pesticides

Six included studies investigated the occurrence of congenital malformation following maternal or paternal exposure to pesticides. These studies either reported non-significant findings (19, 44–46), findings with low precision as indicated by wide confidence intervals (47), or were found to have a high risk of bias (48). Non-occupational exposure to organophosphate pesticides among women conceiving through *in vitro* fertilization were, however, found to be associated with a range of adverse birth outcomes including reduced implantation rate, reduced pregnancy rate, and reduced live birth (49). Women consuming fresh produce with pesticide residues were similarly found to have a reduced incidence of clinical pregnancy and live birth (50). The only study that investigated adverse birth outcomes for paternal exposures to pesticides combined this data with maternal exposure. This study found increased risk of still birth (OR 3.28) and low birthweight, but the latter was reported with wide confidence intervals meaning the estimate should be interpreted with caution (19). Of the three studies examining the association between parental preconception pesticide exposure and childhood illnesses, only one found a significant relationship (51). It identified an increased risk of childhood brain tumors (OR 1.90) for children up to 14 years old associated with either maternal or paternal exposure to pesticides for pest control in the 12 months before conception.

#### 3.4.3 Persistent organic pollutants

Four studies investigated birth outcomes associated with parental exposure to persistent organic pollutants (POP) (52–55) two of which differentiated by parental sex. One examined outcomes on birth rate by sex and found increased male birth rate was associated with a range of organic pollutants although this varied between maternal (from OR 1.09 to OR 1.27) and paternal (from OR 1.06 to OR 1.29) exposure (52). The second study that conducted analysis differentiated by parental sex found birth weight, head circumference and body length outcomes varied based on parental sex, infant sex and the POP type however at least one significant association, encompassing both positive and negative associations, was identified for each POP studied (54). The other two studies investigated low birth weight; the first found no association with maternal intake of fish from areas contaminated with POPs (55), while the second study found high exposure to anti-estrogenic PCBs reduced birth weight but other PCB exposure was non-significant (53).

#### 3.4.4 Organic solvents

Seven studies examined the outcomes associated with parental preconception exposure to organic solvents (18, 56–61). The majority of these focused on cancer-based childhood illness outcomes (leukemia, neuroblastoma, childhood brain tumors) and found risk for both maternal and paternal exposures. One study found increased risk of neuroblastoma associated with maternal occupational exposure to any solvents (OR 2.0), however analysis based on individual solvents resulted in wide confidence intervals and should be interpreted cautiously (61). Similar issues with confidence intervals were noted for a study which reported an increased risk of childhood leukemia associated with maternal, paternal and couple-based occupational exposure to organic solvents for 24 months before conception (58). A second study also found increased childhood leukemia risk and focused solely on paternal occupational exposure to organic solvents 3 months preconception (59). A third study (18) investigated parental occupational exposure to organic solvents. The study identified similar patterns associated with maternal exposure to oxygenated derivatives of hydrocarbons and increased likelihood of childhood leukemia (OR 1.8) and acute lymphocytic leukemia (OR 1.8), and between paternal exposure to polyaromatic hydrocarbons and increased risk of leukemia (OR 1.3), acute lymphocytic leukemia (OR 1.3) and acute lymphoblastic leukemia (OR 1.5).

### 3.5 Other exposures

Twenty-four identified studies reported findings from a range of other general exposure categories published between 1974 and 2019 (see Supplementary Table 6). The exposures in these studies were categorized as radiation (*n* = 9), metals (*n* = 4) and undifferentiated products or compounds (*n* = 14) (see Table 3). They were conducted in United States (*n* = 7), China (*n* = 5), Canada (*n* = 3), Germany (*n* = 2), Mexico (*n* = 2), United Kingdom (*n* = 2), Colombia (*n* = 1), Italy (*n* = 1), Taiwan (*n* = 1) and two international studies conducted in three or more countries. Exposure assessment in this category was overwhelmingly through questionnaire or interview (*n* = 16), with the remainder being personal monitoring devices (*n* = 2), registry or database information (*n* = 2), job matrices (*n* = 2), biospecimens (*n* = 1) and one paper that did not report exposure assessment. Twelve studies included both parents in the exposure population, with an additional eight focused solely on maternal exposures and the remaining four on paternal exposure. The preconception exposure period ranged between 3 months and up to 10 years although four studies did not specify a period, and two studies included any exposure up to the date of conception.

**Table 3:** Outcomes associated with other general exposure categories (*n* = 24).

**Outcome category**	**Study**	**Risk of bias score**	**Preconception exposure period (intensity)**	**Exposure population**	**Environmental exposure**	**Outcomes interested**	**Outcome**	
**Radiation**								
Congenital malformations	Green et al. (62)	7	Up to 6 months (occupational)	Maternal	Cumulative whole body dose occupational exposure to low level ionizing radiation	Chromosomal disorders	NS	
						Congenital malformations with multifactorial etiology	Decreased risk of congenital malformations OR 0.61 (95% CI 0.42, 0.90)	
						Genetic, unspecified	NS	
						Unknown	NS	
						Total congenital malformations	NS	
					Whole body 6 months before conception OR Tritium dose 60 days before conception	Chromosomal disorders	NS	
						Congenital malformations with multifactorial etiology	NS	
						Genetic, unspecified	NS	
						Unknown	NS	
						Total congenital malformations	NS	
	Sever et al. (63)	6	Exposure cumulative to the conception date (occupational)	Maternal and paternal	Exposure to external whole-body penetrating radiation	Neural tube defect	*Maternal exposure* NS	*Paternal exposure* Increased risk of neural tube defect Cases, 24.2 Expected, 13.4 (p = 0.04)
						Other congenital malformations—cleft lip, cleft palate, CHD, tracheoesophageal fistula, pyloric stenosis, hypospadias, congenital dislocation of hip, club foot, limb reduction deformities, down syndrome, multiple system malformation and syndromes	*Maternal exposure* NS	*Paternal exposure* NS
	Ly et al. (46)	4	12-18 months	Paternal	Radiation	Orofacial cleft	NS	
Adverse birth outcomes	Shea et al. (64)	6	12 months (non-occupational)	Paternal	Diagnostic x-rays/Ionizing radiation	Birthweight	NS	
						Gestational age	NS	
						Fetal growth	NS	
Childhood illness	Ou Shu et al. (69)	7	Not specified (occupational)	Maternal and paternal	X-ray exposure	Acute leukemia	*Maternal exposure* None: Reference group 1–5: NS 6–10: NS ≥11: NS	*Paternal exposure* Increased risk of acute leukemia None: Reference group 1–5: NS 6–10: OR 2.4 (95% CI 1.5, 5.0) ≥11: OR 3.9 (95% CI 1.7, 8.6)
						Acute lymphocytic leukemia	*Maternal exposure* None: Reference group 1–5: NS 6–10: NS ≥11: NS	*Paternal exposure* Increased risk of acute lymphocytic leukemia 1–5: Reference group 6–10: OR 1.9 (95% CI 1.2, 2.8) ≥11: OR 2.6 (95% CI 1.5, 4.6)
						Acute non-lymphocytic leukemia	*Maternal exposure* None: Reference group 1–5: NS 6–10: NS ≥11: NS	*Paternal exposure* Increased risk of acute non-lymphocytic leukemia 1–5: Reference group 6–10: NS ≥11: OR 3.7 (95% CI 2.0, 7.0)
	Meinert et al. (68)	6	12 to 24 months (occupational and non-occupational)	Maternal and paternal	Occupational exposure to ionizing radiation (12 months preconception)	Childhood leukemia	*Maternal exposure* NS	*Paternal exposure* No dosimetry: NS Dosimetry: NS
						Childhood non-Hodgkin's lymphomas	*Maternal exposure* NS	*Paternal exposure* No dosimetry: NS Dosimetry: NS
						Childhood solid tumors	*Maternal exposure* NS	*Paternal exposure* No dosimetry: NS Dosimetry: NS
					Diagnostic x-rays	Childhood leukemia	*Maternal exposure* 15-month before conception: NS	*Paternal exposure* Increased risk of childhood leukemia Any site: OR 1.33 (95% CI 1.10, 1.61) Abdomen or intestinal tract: NS
						Childhood non-Hodgkin's lymphomas	*Maternal exposure* 15-month before conception: NS	*Paternal exposure* Any site: NS Abdomen or intestinal tract: NS
						Childhood solid tumors	*Maternal exposure* 15-month before conception: NS	*Paternal exposure* Any site: NS Abdomen or intestinal tract: NS
	Goel et al. (67)	6	12–24 months (non-occupational)	Maternal	Radiation exposure to traditional x-rays (divided into gonadal and non-gonadal regions) and all medical radiation types	Wilms tumor	Total Cases with any x ray 2 years before pregnancy (case 94/control 103) Model 1: NS Model 2: NS Pre-pregnancy 1–2 years before pregnancy (case 74/control 72) Model 1: NS Model 2: NS 1 year before pregnancy (case 43/control 56) Model 1: NS Model 2: NS Gonadal xray Pre-pregnancy (case 11/control 9) Model 1: NS Model 2: NS Non-gonadal xray Pre-pregnancy (case 82/control 89) Model 1: NS Model 2: NS	
	Bunch et al. (66)	5	Not specified (occupational)	Maternal	Ionizing radiation	Leukemia and non-Hodgkin's lymphoma	NS	
						All cancers excluding leukemia and non-Hodgkin's lymphoma	NS	
	Bross and Natarajan (65)	3	Not specified (non-occupational)	Maternal	Radiation other than therapeutic X-rays.	Childhood leukemia (rate at age of diagnosis)	Increased risk of childhood leukemia All kids RR 4.1 (*p* < 0.001) Children aged 1–4 years: RR 3.5 (*p* = 0.002) Children aged 5–9 years: RR 7.4 (*p* = 0.002) Children aged 10–14 years: NS	
**Metals**								
Adverse birth outcomes	Bloom et al. (70)	8	Not specified (non-occupational)	Maternal and paternal	Mercury (blood)	Gestational age (days)	*Maternal exposure* Greater gestational age T1 vs. T2: NS T1 vs. T3: 1.11 (95% CI 0.18, 20.3)	*Paternal exposure* Greater gestational age T1 vs. T2: NS T1 vs. T3: 1.30 (95% CI 0.36, 2.24)
						Birth weight (g)	NS	NS
						Birth length (cm)	*Maternal exposure* Longer birth length T1 vs. T2: NS T1 vs. T3: 1.11 (95% CI 0.18, 2.03)	*Paternal exposure* Longer birth length T1 vs. T2: NS T1 vs. T3: 1.30 (95% CI 0.36, 2.24)
						Head circumference (cm)	NS	NS
						Ponderal index	NS	NS
						Newborn sex	NS	NS
					Arsenic	Gestational age (days)	NS	NS
						Birth weight (g)	NS	*Paternal exposure* Higher birth weight T1 vs. T2: NS T1 vs. T3: 194.71 (95% CI 17.13, 372.30)
						Birth length (cm)	NS	NS
						Head circumference (cm)	NS	NS
						Ponderal index	NS	NS
						Newborn sex	NS	NS
					Cesium	Gestational age (days)	NS	NS
						Birth weight (g)	NS	*Paternal exposure* Lower birth weight T1 vs. T2: NS T1 vs. T3:−237.85 (95% CI−463.04,−12.66)
						Birth length (cm)	NS	NS
						Head circumference (cm)	NS	NS
						Ponderal index	NS	NS
						Newborn sex	NS	NS
					Tungsten (wolfram)	Gestational age (days)	*Maternal exposure* Lower gestational age T1 vs. T2: −1.22 (95% CI −2.19, −0.25) T1 vs. T3: NS	NS
						Birth weight (g)	NS	NS
						Birth length (cm)	*Maternal exposure* Lower birth length T1 vs. T2: −1.22 (95% CI −2.19, −0.25) T1 vs. T3: NS	NS
						Head circumference (cm)	NS	NS
						Ponderal index	NS	NS
						Newborn sex	NS	NS
					Uranium	Gestational age (days)	NS	*Paternal exposure* Lower gestational age T1 vs. T2: −1.10 (95% CI −2.09, −0.11) T1 vs. T3: −1.07 (95% CI −2.07, −0.07)
						Birth weight (g)	NS	*Paternal exposure* Lower birth weight T1 vs. T2: −187.34 (95% CI −366.34, −8.35) T1 vs. T3: NS
						Birth length (cm)	NS	NS
						Head circumference (cm)	NS	NS
						Ponderal index	NS	NS
						Newborn sex	NS	NS
					Zinc	Gestational age (days)	NS	NS
						Birth weight (g)	NS	*Paternal exposure* Lower birth weight T1 vs. T2: NS T1 vs. T3: −209.08 (95% CI −417.40, −0.77)
						Birth length (cm)	NS	NS
						Head circumference (cm)	NS	NS
						Ponderal index	NS	NS
						Newborn sex	NS	NS
					Other heavy metals: Barium Cadmium Chromium Cobalt Copper Lead (blood) Molybdenum Selenium Thallium Tin Antimony	Gestational age (days)	NS	NS
						Birth weight (g)	NS	NS
						Birth length (cm)	NS	NS
						Head circumference (cm)	NS	NS
						Ponderal index	NS	NS
						Newborn sex	NS	NS
	Ly et al. (46)	4	12-18 months (occupational)	Paternal	Lead	Orofacial cleft	NS	
Childhood illnesses	Miligi et al. (18)	6	12 months (occupational)	Maternal and paternal	Chromium	Leukemia	*Maternal exposure* NS	*Paternal exposure* Increased risk of leukemia OR 2.1 (95% CI 1.1, 4.2)
						Acute lymphocytic leukemia	*Maternal exposure* NS	*Paternal exposure* Increased risk of acute lymphocytic leukemia OR 2.0 (95% CI 1.0, 4.2)
					Nickel	Leukemia	*Maternal exposure* N/A	*Paternal exposure* NS
						Acute lymphocytic leukemia	*Maternal exposure* N/A	*Paternal exposure* NS
					Lead	Leukemia	*Maternal exposure* NS	*Paternal exposure* Increased risk of leukemia OR 1.7 (95% CI 1.1, 2.7)
						Acute lymphocytic leukemia	*Maternal exposure* NS	*Paternal exposure* NS
	Olshan et al. (60)	4	Any time prior to pregnancy	Paternal	Lead	Wilms' tumor	NS	
					Boron	Wilms' tumor	NS	
**Undifferentiated products or compounds**								
Congenital malformations	Chen et al. (77)	6	12 months (not specified)	Maternal	Toxic substance	Birth defects	Increased birth defects OR 5.37 (3.60, 7.99)	
	Liu et al. (73)	4	12–18 months (occupational)	Maternal	Interior decoration or oil paint odor	Macrosomia	Increased macrosomia OR 1.297 (1.133, 1.484)	
	Ly et al. (46)	4	12–18 months (occupational)	Paternal	Industrial chemical	Orofacial cleft	Reduced risk of orofacial cleft OR 0.51 (95% CI 0.30, 0.85)	
					Chemical waste	Orofacial cleft	NS	
Adverse birth outcomes	Qu et al. (19)	7	Up to 6 months (not specified)	Maternal and paternal	new decoration/renovation	Stillbirth	*Maternal exposure* NS	*Paternal exposure:* Increased stillbirth OR: 1.64 (1.01, 2.69)
	Sung et al. (75)	6	3 months (occupational), up to 10 years	Paternal	Electronics factory exposure	Infant mortality < 12 months	Increased risk of infant mortality *Occupational exposure as a non-manager* >10 years: RR 5.06 (95% CI 2.33, 11.00) 1–10 years: RR 2.81 (95% CI 1.44, 5.51) < 1 year: NS *Occupational exposure as a manager* NS	
	Ly et al. (46)	4	12–18 months (occupational)	Paternal	Industrial chemical	Orofacial cleft	Reduced risk of orofacial cleft OR 0.51 (95% CI 0.30, 0.85)	
					Chemical waste	Orofacial cleft	NS	
Childhood illness	Liu et al. (74)	7	3 months (non-occupational)	Maternal	Less than 1 month since housing renovation defined as use of marble, plywood, laminated particle board, carpets, ceramic tile, oil-based paint, latex, acrylic or wallpaper.	Congenital heart disease	Increased congenital heart disease OR: 2.38 (1.03, 5.48)	
	Slater et al. (71)	7	1 month (non-occupational)	Maternal	*Household chemical exposures* Paints, stains, lacquers Petroleum products	Acute lymphoblastic leukemia	OR: NS	
						Acute myeloid leukemia	OR: NS	
						Mixed lineage leukemia gene rearrangement present	OR: NS	
						No mixed lineage leukemia gene rearrangement present	OR: NS	
	Perez-Saldivar et al. (78)	7	At least 6 months in the 2 years preconception (occupational)	Paternal	At least one occupation considered to have a high exposure to ‘carcinogenic agents'	ALL or AML	NS	
	Ou Shu et al. (69)	7	Not specified (occupational)	Maternal and paternal	Solvents, degreasers, or cleaning agents	Acute lymphocytic leukemia	*Maternal exposure* Increased risk of acute lymphocytic leukemia OR 1.8 (95% CI: 1.3, 2.5)	
					Plastic materials	Acute lymphocytic leukemia	*Maternal exposure* NS	
					Paints or thinners	Acute lymphocytic leukemia	*Maternal exposure* Increased risk of acute lymphocytic leukemia OR 1.6 (95% CI: 1.2, 2.2)	
					Oil or coal products	Acute lymphocytic leukemia	*Maternal exposure* NS	
					Other (epoxy resin, fomaldehyde, glues, exhaust, fuels)	Acute lymphocytic leukemia	*Maternal exposure* Increased risk of acute lymphocytic leukemia OR 1.3 (95% CI: 1.0, 1.7)	
	Castro-Jimenez and Orozco-Vargas (58)	6	24 months (occupational) l, maternal and paternal,	Maternal and paternal	Mineral oils	Acute lymphocytic leukemia	*Maternal exposure* Increased risk of acute lymphocytic leukemia 6.70 (1.51, 29.69)	*Paternal exposure* NS	*Both parental exposure* Increased risk of acute lymphocytic leukemia 11.26 (2.80, 45.24)
					Coal-tar pitches	Acute lymphocytic leukemia	*Maternal exposure* NS	
	Nie et al. (72)	6	3 months preconception (not specified)	Paternal	Chemicals	Congenital heart disease	Increased congenital heart disease OR 26.78 (95% CI 9.85, 72.83)	
	Svanes et al. (76)	6	>10 years preconception (occupational)	Paternal	Welding or metal fumes	Early-onset asthma,	Increased risk of early-onset asthma OR: 1.64 (95% CI 1.25, 2.15)	
						Early-onset allergic asthma	NS	
						Early-onset non allergic asthma	Increased risk of early-onset non-allergic asthma OR: 1.80 (95% CI 1.29, 2.50)	
						Later-onset asthma	NS	
	Schüz et al. (20)	6	12 months (occupational)	Maternal and paternal	Solvents	Acute lymphocytic leukemia	*Maternal exposure* NS	
					Paints or lacquers	Acute lymphocytic leukemia	*Maternal exposure* Increased risk of acute lymphocytic leukemia OR 1.6 (95% CI 1.1, 2.4)	
					Oil products	Acute lymphocytic leukemia	*Maternal exposure* NS	
					Plastic or resin fumes	Acute lymphocytic leukemia	*Maternal exposure* NS	
					Metal melting	Acute lymphocytic leukemia	*Maternal exposure* NS	
	Miligi et al. (18)	6	12 months (occupational)	Maternal and paternal	Mineral oils	Leukemia	*Maternal exposure* OR: NS	
						Acute lymphocytic leukemia	*Maternal exposure* OR: NS	
	Pérez-Saldivar (79)	5	Unspecified preconception period (occupational and non-occupational)	Maternal and paternal	Exposure to carcinogens at home (use of petroleum products at home like oil paint, solvents, lacquers, wood varnishes, insecticides, pesticides, etc)	Acute leukemia	Increased acute leukemia Paternal exposure: NS Maternal exposure: OR 2.69 (95% CI 1.59, 4.53)	
					Overall exposure to carcinogens	Acute leukemia	NS	

#### 3.5.1 Radiation

Nine included studies investigated the association between radiation exposure and congenital malformations (46, 62, 63), adverse birth outcomes (64), and childhood illness (65–69). The association between radiation exposure and congenital malformations or adverse birth outcomes were commonly non-significant, except for occupational exposure to whole body ionizing radiation which was associated with a decreased risk of congenital malformations with multifactorial etiology (maternal exposure) (OR 0.61) and increased risk of neural tube defect (paternal exposure) (*p* = 0.04). The results of studies examining the associations between either maternal or paternal exposure to radiation and childhood illness only found statistically significant outcomes for paternal exposure to x-rays and risk of leukemia (68, 69), except for one study published in 1974 which also reported an increased risk of leukemia based on maternal exposure to unspecified radiation other than x-rays (65). Neither maternal nor paternal occupational exposure to ionizing radiation was identified as having a significant effect on leukemia, lymphoma, or solid tumors (66, 68).

#### 3.5.2 Metals

The relationship between parental preconception exposure to metals and infant and child health outcomes was investigated in four studies (18, 46, 60, 70) that demonstrated some association with leukemia, low birthweight and lower gestational age, however these associations for individual metals varied in significance between studies for different studies as such should be interpreted with caution. For example, one of the studies identified a range of adverse birth outcomes were examined in one study and found six of the seventeen metals investigated had at least one statistically significant outcome for either maternal or paternal exposures. However, the direction of the relationship was often not consistent; some metals (e.g., arsenic, mercury) reported a positive association with birth length, birth weight or gestational age while others (e.g., tungsten, uranium, zinc) reported a negative association with these outcomes (70). More consistent outcomes were identified for paternal occupational exposure to metals in the 12 months preconception, in which chromium or lead were found to increase risk of leukemia in children up to 10 years old.

#### 3.5.3 Undifferentiated products or compounds

The risk of adverse outcomes associated with parental preconception exposure to products or compounds that may contain substances from several categories were investigated in 15 studies. These included paint or thinners (20, 69, 71), unspecified chemicals (46, 72), housing renovations or interior decoration (19, 73, 74), plastic materials (20, 69), electronic factory exposures or welding and metal fumes (20, 75, 76), coal products (58, 69), and general descriptions of “toxic substances” (77) or “carcinogens” (78, 79) among others. Two (46, 73) of the three studies investigating congenital malformations had a high risk of bias, while the third (77) found an increased risk of unspecified birth defects associated with maternal exposure to “toxic substances” in the 12 months before pregnancy. Paternal exposure to new decoration or renovation up to 6 months preconception was associated with increased stillbirth (OR 1.64), but the study did not find a similar result for maternal exposure (19). Seven of the included studies focused on childhood leukemia as the outcome of their analysis, and the results varied based on exposure (18, 20, 58, 69, 71, 78, 79). An increased risk of acute lymphocytic leukemia was associated with maternal exposure to solvents, degreasers, or cleaning agents (OR 1.8) (69), paints or thinners (OR 1.6) (20, 69), and other various compounds (69, 78). Other maternal and paternal preconception exposures examined for risk of leukemia were either non-significant or had low precision. Paternal occupational exposure to an electronic factory environment as a non-manager was found to increase risk of infant mortality if the exposure had occurred at any point between 1 and 10 years before the child was conceived (RR 2.81) (75). The risk ratio was found to be even greater for those working more than 10 years, but the confidence interval was very wide and so should be interpreted cautiously. Paternal exposure to welding or metal fumes in the 10 years or more before conception was associated with an increased risk of early-onset non-allergic asthma in the child by 2 years old (79). Mother's exposure to housing renovations (OR 2.38) in the 3 months before pregnancy was reported to increase the risk of congenital heart disease in their child (74).

## 4 Discussion

This review presents the first critical summary of observational research examining the relationship between maternal or paternal preconception environmental exposures, and child health outcomes. The review demonstrates that this topic has received research attention for almost 50 years and consistent attention since the late 1990s. It also shows the breadth of exposures that encompass environmental health as a field. Environmental exposures are often not prioritized in health advice or public health guidelines for preconception care (80–84), yet every environmental exposure category covered by this review demonstrated at least one adverse child health outcome. The review also highlights the range of preconception exposures viewed as potentially relevant by environmental health researchers and show that human exposures occur in both occupational and domestic environments, as well as demonstrating the ubiquity of co-occurring environmental exposures. These trends in the review findings emphasize the complexity of environmental health considerations in the context of preconception health and the need for more targeted research on this topic through a coordinated research agenda. Future literature reviews, for example, can be informed by the topics identified through our broad-scoped review and provide targeted follow up by focusing on specific exposure and outcome categories. Such work will be able to further advance evidence synthesis on this topic (e.g., meta-analysis, umbrella reviews) as new research continues to emerge.

The review findings suggest parental and child sex may be an important modifier of the association between preconception environmental exposures and child health outcomes. The study of exposures differed significantly by parental sex as most of the identified research only focused on exposure in one parent, primarily the mother. This imbalance was particularly noticeable in exposure categories such as air pollutants and ambient temperature research. It is possible this trend is reflective of the challenges of collecting reliable data on paternal health given men are less likely to attend antenatal or child health appointments where study participants may be recruited (85, 86), however it is unlikely that recruitment through clinical settings would have affected the characteristics of most of the established cohort studies represented in this review. As such, it is also possible that researchers' historical interest in exposures in the female parent during pregnancy (85) has extended to preconception research. It is also possible that limitations in datasets that capture paternal preconception clinical data preclude meaningful investigation of paternal exposures, as has been identified in paternal preconception research beyond environmental health (87). Yet, the evidence provided by this review suggests both parents' exposures may impact on child health albeit with different outcomes. For example, x-rays appear to have a differing risk profile for maternal compared with paternal exposure in the preconception period, whereby paternal exposure may increase risk of leukemia. The body of research for POP examined parental sex-specific effects more than other environmental exposures and reports various outcomes dependent on the sex of the parent and the outcome of interest. It is also worth noting that some differences in outcomes based on child sex were evident in the studies investigating POPs [compounds known to affect hormone regulation and expression (88)] but as this was the only category that differentiated by child sex it is difficult to say whether this is a unique characteristic of POPs or has wider implications. Beyond these modifying factors, none of the included papers attempted to assess the potential mechanisms.

The review also identified the importance of considering both occupational and non-occupational exposures when studying parental preconception environmental exposures. While occupational exposures of substances such as solvents have received early attention in the timeline of research for this topic [e.g., Cassidy et al. (57) and Olshan et al. (60)], more contemporary studies included in this review indicates adverse neonatal outcomes are fairly consistent across various forms of exposure. For example, domestic, occupational, and dietary exposure to pesticides were all associated with adverse child health outcomes, demonstrating that effects are apparent even at the lower levels encountered in domestic settings. For this reason, pesticide exposure is an area of concern and could be targeted in preconception health care interventions. Existing guidelines highlight the importance of safety when exposed to pesticides in the preconception period, but these guidelines commonly focus on occupational exposure (89) with only more recent attention given to domestic exposure (90). Other categories, such as solvents, were only studied for occupational exposure, where exposures typically occur at higher concentrations for workers and their families (91), and focused on cancer-based outcomes. However, some preconception populations may spend a higher proportion of their day in a domestic rather than occupational setting (e.g., parents who are primary carers for young children) and exposures that occur in occupational settings can also occur in domestic settings (92).

### 4.1 Future research

While this review provides a comprehensive overview of the state of the science with regards to preconception environmental exposures and child health outcomes, it also identifies a number of critical gaps that require urgent research attention. Given the changing heat patterns already present and expected to worsen through global climate change (93), future research needs to extend on the study of ambient temperature identified through this review to include both maternal and paternal populations. New research also needs to examine the effects of exposure to hazards associated with decoration or renovation in the preconception period beyond the limited attention reflected in the findings of this review. “Nesting” is commonly discussed in pregnancy forums as a behavior couples (particularly women) may undertake in preparation for pregnancy (94), yet home renovation results in exposure to solvents and other chemicals (95) that this review identifies as problematic. The combined effects of these exposures at non-occupational concentrations in a domestic setting requires urgent attention. Particularly given “home renovators” may not follow the same safety precautions as an individual exposed to these compounds through their work (95). Contemporary research is needed to examine outcomes of preconception x-ray exposures, as the evidence available is dated. Equally, there is a clear need for additional research that focuses on both male and female exposures. Several studies included in this review identified adverse outcomes associated with male exposures, but too often male exposures were not measured. For this reason, it is possible that the focus on female preconception exposures is masking the effects of male exposures. Lastly, future research in this field should begin to consider and evaluate the potential causal mechanisms during this important time period. Causal inference methods are further advanced in other environmental health topics, such as air pollution research (96), and these techniques should be applied to preconception environmental health works.

### 4.2 Review limitations

These results of this review should be considered within the context of its limitations. Firstly, some of the studies showed low precision, demonstrated by their wide confidence intervals. Similarly, numerous studies reported small effect sizes, making it difficult to find relationships when the outcome is rare. While evidence across studies affords more confidence in an association, future research needs larger sample sizes to address these issues. With the advent of data linkage, it may be possible to better explore outcomes across larger datasets or multi-cohort studies. The definition of the preconception period was often unclear or absent in some of the included studies. This limits the transferability of the findings into clinical recommendations. Studies were excluded if they overlapped the preconception and pregnancy period, such as periconception, as this overlap prohibited the ability to differentiate the contribution between preconception and pregnancy exposures. As a result, the pool of evidence pertaining to the research question was reduced. Future research should distinguish between these two exposure periods more effectively to allow a clearer evidence-base to emerge. However, it should also be acknowledged that several included studies relied on secondary analysis of hospital data and that such data sources do not readily allow for distinction between exposure periods.

Due to the numerous exposures and outcomes identified through the review, in line with the broad field of environmental health, a meta-synthesis of study findings was not possible. While this also adds to the strength of the review due to the broad-based knowledge captured in the included studies, it limits comprehensive synthesis of findings. The breadth of environmental health research areas may also mean some relevant studies may have been missed. However, the review employed a comprehensive search strategy informed by a research librarian and as such it is not expected that many studies would have been overlooked. The was also a timelapse between the search and the publication of the article, which may mean that any more recent studies were not included. However, as the original search was conducted without date restrictions and identified more than 60 studies published over 50 years, the review still provides a comprehensive overview of the science of this topic to date. The review is also strengthened by the involvement of two researchers at all stages of article selection, critical appraisal, and data extraction. Furthermore, no language restrictions were applied to the review.

### 4.3 Conclusion

This review indicates the research related to the outcomes of environmental preconception exposures has developed over 50 years. While some topics have received focused attention from research teams in that time, most studies appear to standalone and have not continued to develop as part of wider research programs. As governments are beginning to develop policy and health system responses to preconception health risks in the population, they will require more comprehensive information about preconception environmental exposures and their associated outcomes for mother and child. For this reason, a research agenda for environmental preconception health exposures and outcomes that supports a more coordinated, targeted, and strategic effort is urgently needed. Researchers must move beyond rare outcomes and the “opportunistic” use of databases to more thoughtful and progressive cohorts. Future research needs to build on advances in reproductive environmental epidemiology such as causal inference methods, exposome analysis, and the inclusion of—omics data. However, even with the constraints of these issues, this review suggests there is growing evidence of adverse offspring outcomes associated with maternal and paternal environmental exposures during the pre-conception period. As such, this topic requires greater focus by the research community, public health agencies and clinicians to reduce the prevalence of non-communicable disease.

## Data Availability

The original contributions presented in the study are included in the article/Supplementary material, further inquiries can be directed to the corresponding author.
