# Supplementary material for: The influence of environmental exposures during the preconception period on offspring outcomes: a systematic review
Source: Front Public Health. 2025 Dec 17;13:1633266. doi: 10.3389/fpubh.2025.1633266 (PMC12753933; doi:10.3389/fpubh.2025.1633266)
Supplement: Supplementary file 1 [file Table_1.docx]

**Supplementary Table 1: Generic search terms by category.**

| **PICO** | **Key words** | **MeSH Terms** |
| --- | --- | --- |
|  |  |  |
| **Population**  *Reproductive-aged men or women that are self-identified or identified by the researchers as being in the preconception period and as being the reproductive parent of the child for which the outcome(s) is/are reported.* | preconception  pre-conception  periconceptional  peri-conceptional  pre-pregnancy  prepregnancy  interconception | preconception care |
| **Exposure**  *Any environmental hazard, such as air pollutants, heavy metals, or chemical exposures* | ”environmental health”  “air quality”  “air pollution”  chemical  plastics  plasticizers  “heavy metal”  pesticides  “occupational exposure”  “disinfection by-products  “endocrine disrupting chemicals”  “persistent organic pollutants” | Environment  Environmental pollution  Environmental exposure |
| **Outcome(s)**  *Positive or adverse neonatal, infant or childhood outcomes, or life course offspring outcomes* |  |  |
| *Pregnancy outcome:* | fertility  pregnancy | infertility  exp pregnancy outcome  exp pregnancy complications |
| *Infant or child or life course outcome:* | infant outcome  child outcome  life course  offspring outcome | exp fetal development  perinatal death  child mortality  exp congenital abnormalities  exp fetal diseases  exp infant newborn diseases  noncommunicable diseases |
| *All key words and MeSH Terms in columns will be combined with Boolean operator ‘OR’ and each search domain ‘Population’, ‘Exposure’, ‘Outcome’ will be combined with Boolean operator ‘AND’* | | |

**Supplementary Table 2: Risk of Bias assessment for all studies**

| **Case control studies** | **Is the case definition adequate?** | **Representativeness of the cases** | **Selection of controls** | **Definition of controls** | **Comparability of cases and controls on the basis of the design or analysis** | **Ascertainment of exposure** | **Same method of ascertainment for cases and controls** | **Non-response rate** | **Score** |
| --- | --- | --- | --- | --- | --- | --- | --- | --- | --- |
| Addissie, et al (2020) [45] | 0 | 0 | 1 | 0 | 2 | 0 | 1 | 0 | 4 |
| Aguilar-Garduño, et al (2010) [56] | 1 | 1 | 1 | 1 | 1 | 0 | 1 | 0 | 6 |
| Bross and Natarajan (1974) [65] | 1 | 0 | 0 | 1 | 1 | 0 | 1 | 0 | 3 |
| Bunch, et al (2009) [66] | 0 | 1 | 1 | 0 | 1 | 0 | 1 | 0 | 5 |
| Cassidy, et al (1989) [57] | 1 | 1 | 1 | 1 | 1 | 0 | 1 | 1 | 6 |
| Castro-Jimenez, et al (2011) [58] | 1 | 1 | 1 | 1 | 1 | 0 | 1 | 0 | 6 |
| Chen, et al (2008) [77] | 0 | 1 | 1 | 1 | 1 | 0 | 1 | 1 | 6 |
| Pérez-Saldivar, et al (2016) [79] | 1 | 1 | 0 | 1 | 1 | 0 | 1 | 0 | 5 |
| Gashaw, et al (2021) [47] | 1 | 1 | 0 | 1 | 1 | 0 | 1 | 1 | 6 |
| Goel, et al (2009) [67] | 1 | 1 | 1 | 0 | 1 | 0 | 1 | 1 | 6 |
| Green, et al (1997) [62] | 1 | 1 | 1 | 0 | 2 | 1 | 1 | 0 | 7 |
| Greenop, et al (2013) [51] | 1 | 1 | 1 | 1 | 1 | 0 | 1 | 1 | 7 |
| Huang, et al (2020) [21] | 0 | 1 | 1 | 1 | 1 | 1 | 1 | 1 | 7 |
| Jiang, et al (2021) [22] | 0 | 1 | 0 | 1 | 1 | 1 | 1 | 1 | 6 |
| Kalkbrenner, et al (2015) [37] | 1 | 1 | 1 | 1 | 1 | 1 | 1 | 1 | 8 |
| Lacasaña, et al (2006) [44] | 1 | 1 | 1 | 1 | 1 | 0 | 1 | 1 | 7 |
| Liu, et al (2013) [74] | 1 | 1 | 1 | 1 | 1 | 0 | 1 | 1 | 7 |
| Liu, et al (2020) [23] | 1 | 1 | 1 | 1 | 1 | 1 | 1 | 1 | 8 |
| Ly, et al (2017) [46] | 1 | 1 | 1 | 1 | 1 | 0 | 1 | 0 | 4 |
| Meinert, et al (1999) [68] | 1 | 1 | 1 | 1 | 1 | 0 | 1 | 0 | 6 |
| Mekonnen, et al (2021) [32] | 0 | 1 | 0 | 1 | 1 | 1 | 1 | 1 | 6 |
| Mendola, et al (2016) [33] | 1 | 1 | 0 | 1 | 1 | 1 | 1 | 1 | 7 |
| Miligi, et al (2013) [18] | 1 | 1 | 1 | 0 | 1 | 1 | 1 | 0 | 6 |
| Nie, et al (2013) [72] | 1 | 1 | 0 | 1 | 1 | 0 | 1 | 1 | 6 |
| Nobles, et al (2019) [34] | 1 | 1 | 0 | 1 | 1 | 1 | 1 | 1 | 7 |
| Olshan, et al (1990) [60] | 0 | 1 | 0 | 1 | 1 | 0 | 1 | 0 | 4 |
| Ou Shu, et al (1988) [69] | 1 | 1 | 1 | 1 | 2 | 0 | 1 | 0 | 7 |
| Parodi, et al (2014) [61] | 1 | 1 | 1 | 1 | 1 | 0 | 1 | 1 | 7 |
| Perez-Saldivar, et al (2008) [78] | 1 | 1 | 1 | 1 | 1 | 0 | 1 | 1 | 7 |
| Ren, et al (2018) [24] | 0 | 1 | 0 | 1 | 1 | 1 | 1 | 1 | 6 |
| Schüz, et al (2000) [20] | 1 | 1 | 1 | 1 | 1 | 0 | 1 | 0 | 6 |
| Sever, et al (1988) [63] | 1 | 1 | 0 | 1 | 1 | 1 | 1 | 0 | 6 |
| Slater, et al (2011) [71] | 1 | 1 | 1 | 1 | 1 | 0 | 1 | 1 | 7 |
| Talbott, et al (2015) [39] | 1 | 1 | 1 | 1 | 1 | 1 | 1 | 1 | 8 |
| Weisskopf, et al (2005) [55] | 0 | 0 | 1 | 0 | 1 | 0 | 1 | 1 | 4 |
| Zhang, et al (2020) [26] | 0 | 1 | 0 | 1 | 1 | 0 | 1 | 1 | 5 |
| **Cohort studies** | **Representativeness of the exposed cohort** | **Selection of the non-exposed cohort** | **Ascertainment of exposure** | **Outcome not present at start of study** | **Comparability of cohorts on the basis of the design or analysis** | **Assessment of outcome** | **Follow-up was long enough for outcomes to occur** | **Adequacy of follow up of cohorts** | **Score** |
| Bae, et al (2018) [52] | 1 | N/A | 1 | 1 | 2 | 0 | 1 | 0 | **6** |
| Bloom, et al (2015) [70] | 1 | N/A | 1 | 1 | 2 | 1 | 1 | 1 | **8** |
| Yao, et al (2016) [25] | 1 | N/A | 1 | 1 | 2 | 1 | 1 | 1 | **8** |
| Chiu, et al (2017) [50] | 1 | N/A | 0 | 1 | 2 | 1 | 1 | 1 | **7** |
| Zhang, et al (2021) [41] | 1 | N/A | 1 | 1 | 2 | 1 | 1 | 0 | **7** |
| Jo, et al (2019) [36] | 1 | N/A | 1 | 0 | 2 | 1 | 1 | 1 | **7** |
| Zhang, et al (2020) [40] | 1 | N/A | 1 | 1 | 2 | 1 | 1 | 0 | **7** |
| Ha, et al (2017) [28] | 1 | N/A | 1 | 0 | 2 | 1 | 0 | 0 | **5** |
| Ha, et al (2017) [29] | 1 | N/A | 1 | 0 | 2 | 1 | 0 | 0 | **5** |
| Ha, et al (2017) [30] | 1 | N/A | 1 | 0 | 2 | 1 | 0 | 0 | **5** |
| Han, et al (2020) [31] | 1 | N/A | 1 | 1 | 2 | 1 | 1 | 1 | **8** |
| Heck, et al (2019) [59] | 1 | 1 | 1 | 0 | 2 | 1 | 0 | 0 | **6** |
| Hu, et al (2020) [49] | 1 | N/A | 1 | 1 | 2 | 1 | 1 | 1 | **8** |
| Qu, et al (2019) [19] | 1 | 1 | 0 | 1 | 2 | 1 | 1 | 0 | **7** |
| Kuiper, et al (2020) [17] | 1 | N/A | 1 | 0 | 2 | 0 | 0 | 0 | **4** |
| Shea, et al (1997) [64] | 1 | 1 | 0 | 1 | 2 | 0 | 1 | 0 | **6** |
| Liu, et al (2017) [73] | 1 | N/A | 1 | 1 | 2 | 1 | 1 | 1 | **8** |
| Mao, et al (2017) [38] | 1 | N/A | 1 | 1 | 1 | 0 | 1 | 1 | **6** |
| Murphy, et al (2010) [53] | 1 | N/A | 1 | 1 | 1 | 0 | 1 | 1 | **6** |
| Mustieles, et al (2018) [42] | 1 | N/A | 1 | 1 | 2 | 1 | 1 | 0 | **7** |
| Robledo, et al (2015) [54] | 1 | N/A | 1 | 1 | 2 | 0 | 1 | 0 | **6** |
| Seeni, et al (2018) [35] | 1 | N/A | 1 | 0 | 2 | 1 | 0 | 0 | **5** |
| Svanes, et al (2016) [76] | 1 | 1 | 0 | 0 | 2 | 0 | 1 | 1 | **6** |
| Smarr, et al (2015) [43] | 1 | N/A | 1 | 1 | 2 | 0 | 1 | 1 | **7** |
| Sung, et al (2009) [75] | 1 | 1 | 1 | 0 | 2 | 1 | 0 | 0 | **6** |
| Weselak, et al (2008) [48] | 0 | N/A | 0 | 0 | 2 | 0 | 1 | 0 | **3** |
| Zhu, et al (2015) [27] | 1 | N/A | 1 | 0 | 2 | 1 | 0 | 0 | **5** |

**Supplementary Table 3: Characteristics of included studies investigating outcomes associated with ambient air pollution (n = 21) and ambient temperature (n=3)**

| **Study** | **Country** | **Study design** | **Date** | **Total participants** | **Population** | **Preconception period** | **Exposure assessment method** | **Exposure population** | | **Age of child** |
| --- | --- | --- | --- | --- | --- | --- | --- | --- | --- | --- |
|  |  |  |  |  |  |  |  | **Maternal** | **Paternal** |  |
| Yao, et al (2016) [25] | China | cross-sectional | Jan 2010 - Dec 2012 | 16,332 | The local Maternity and Child Care Centre. Birth defects specific data was collected from the Birth Defects Monitoring System | 3 months | Local environmental monitoring center | √ | x | Newborns |
| Ha, et al (2017) [30] | United States | cross-sectional | 2002 - 2008 | 220,572 | Existing cohort known as the Air Quality and Reproductive Health study | 3 months | Meteorological models | √ | x | Neonate |
| Han, et al (2020) [31] | China | prospective | Jan 2014 - Dec 2015 | 1,507 | Hospitals affiliated with Nanjing Medical University | 3 months | Local monitoring stations | √ | x | Not specified |
| Huang, et al (2020) [21] | Taiwan | case-control | 2007 - 2014 | 22,000 | Registry data (the Taiwanese Birth Registry database) | 3 months | Geospatial Interpolation method - Kriging | √ | x | Not specified |
| Jiang, et al (2021) [22] | China | case-control | 2010 - 2015 | 11,150 | Registry data (the Maternal and Child Health Certificate Registry of Liaoning Province) | 3 months | Local monitoring stations | √ | x | Not specified |
| Jo, et al (2019) [36] | United States | cross-sectional | Jan 1999 - Dec 2009 | 246,420 | Electronic medical records from Kaiser Permanente Southern California (KPSC) (health provider) | 3 months | EPA regional air quality monitoring network | √ | x | Age 1 to 5 |
| Kalkbrenner, et al (2015) [37] | United States | case-control | 1994 - 2000 | 15,645 | Routine records (case data from autism surveillance systems with birth records) | 2.6 months | Geospatial interpolation method – temporal resolution | √ | x | Age 8 |
| Kuiper, et al (2020) [17] | Norway and Sweden | cross-sectional | 1975 - unclear | 1,949 | Existing cohort known as the Respiratory Health in Northern Europe, Spain and Australia (RHINESSA) generation study | 0 to 18 years | Land use regression models | √ | √ | Not specified |
| Liu, et al (2020) [23] | China | case-control | 2010 - 2015 | 11,036 | Routine records | 1 month; 2 months; 3 months | Local monitoring stations | √ | x | >Age 1 |
| Mao, et al (2017) [38] | United States | prospective | 2003 - 2012 | 1,446 | Existing cohort known as the Boston Birth Cohort | 3 months | EPA air quality monitoring stations | √ | x | Age 2 to 9 |
| Mekonnen, et al (2021) [32] | United States | case-control | 2007 - 2011 | 953,951 | Routine records (obtained from the Birth Statistical Master files at the California Department of Public Health in six California counties of Alameda, Contra Costa, Fresno, Los Angeles, San Diego, and San Francisco) | 3 months | National, state and local monitoring stations | √ | x | Not specified |
| Mendola, et al (2016) [33] | United States | case-control | 2005 - 2007 | 204,175 women  (223,502 pregnancies) | Existing cohort known as the Consortium on Safe Labor | 3 months | Modified EPA Community Multiscale Air Quality models | √ | x | Not specified |
| Miligi, et al (2013) [18] | Italy | case-control | 1998 - 2001 | 1,824 | Cases: SETIL study (participants recruited through paediatric oncology centres)  Controls: chosen randomly from the local population in each region using the national health service records | 12 months | Interviewer administered questionnaire | √ | √ | Age 0 to 10 |
| Nobles, et al (2019) [34] | United States | case-control | 2002 - 2010 | 50,005 women  (112,203 singleton births) | An existing study known as the Eunice Kennedy Shriver National Institute of Child Health and Human Development (NICHD) Consecutive Pregnancy Study | 3 months | Modified EPA Community Multiscale Air Quality models | √ | x | Not specified |
| Qu, et al (2019) [19] | China | prospective | 2010 - 2013 | 7,299 | National Free Preconception Health Examination Project | Up to 6 months | Self-report questionnaire | √ | √ | At birth |
| Ren, et al (2018) [24] | United States | case-control | 2006 - 2010 | 548,863 | Routine records (Ohio Department of Health live birth records). | 1 to 2 months | EPA air quality monitoring stations | √ | x | Not specified |
| Schüz, et al (2000) [20] | Germany | Other: Pooled analysis from three case-control studies | 1992 - 1997 | 4,100 | (1) a case-control study on childhood cancer in the Northwestern part of Germany; (2) a case-control study on childhood leukaemia and childhood lymphoma in the vicinity of German nuclear installations and selected control regions; (3) a case-control study on childhood cancer covering the entire region of Western Germany. | 12 months | Self-report questionnaire | √ | √ | Below age 15 |
| Seeni, et al (2018) [35] | United States | ecological | Jan 2002 - Dec 2008 | 223,375 | Electronic medical records from 12 clinical sites (15 hospital referral regions) across the USA (from the Consortium on Safe Labor cohort) | 3 months | Modified EPA Community Multiscale Air Quality models | √ | x | Newborn |
| Talbott, et al (2015) [39] | United States | case-control | Jan 2005 - Dec 2009 | 430 | Cases: specialty autism clinics and treatment centres, local pediatric and family medicine practices, and the Intermediate Units of the Pennsylvania School System  Controls: a random selection of births from the Pennsylvania Department of Health | 3 months | Land use regression models | √ | x | Not specified |
| Zhang, et al (2020) [26] | China | case-control | Jan 2010 - Dec 2015 | 11,150 | Maternal and Child Health Certificate Registry of Liaoning Province. | 3 months | Regional air quality monitoring stations | √ | x | Age 0 to 1 |
| Zhu, et al (2015) [27] | United States | cross-sectional | Jan 2002 - Dec 2008 | 188,102 | Electronic records in the Consortium on Safe Labor (12 clinical centres across 9 American College of Obstetricians and Gynecologists US districts) | 3 months | Modified EPA Community Multiscale Air Quality models | √ | x | Newborn |

**Supplementary Table 4: Characteristics of included studies investigating outcomes associated with ambient temperature (n = 3)**

| **Study** | **Country** | **Study design** | **Date** | **Total participants** | **Population** | **Preconception period** | **Exposure population** | | **Age of child** | **Exposure assessment method** |
| --- | --- | --- | --- | --- | --- | --- | --- | --- | --- | --- |
| Ha, et al (2017) [28] | United States | cross-sectional | 2002 - 2008 | 223,375 | Existing cohort known as the Air Quality and Reproductive Health study | 3 months | √ | x | Neonate | Meteorological models |
| Ha, et al (2017) [30] | United States | cross-sectional | 2002 - 2008 | 220,572 | Existing cohort known as the Air Quality and Reproductive Health study | 3 months | √ | x | Neonate | Meteorological models |
| Ha, et al (2017) [29] | United States | cross-sectional | 2002 - 2008 | 223,375 | Existing cohort known as the Air Quality and Reproductive Health study | 3 months | √ | x | Not specified | Meteorological models |

**Supplementary Table 5: Characteristics of included studies investigating outcomes associated with exposure to chemicals (n=26)**

| **Study** | **Country** | **Study design** | **Date** | **Total participants** | **Population** | **Preconception period** | **Exposure assessment method** | **Exposure population** | | **Age of child** |
| --- | --- | --- | --- | --- | --- | --- | --- | --- | --- | --- |
|  |  |  |  |  |  |  |  | **Maternal** | **Paternal** |  |
| Addissie, et al (2020) [45] | United States | case-control | Mar 2016 - Feb 2019 | 147 | Existing studies and registries combined with community recruitment through advertisement in websites and recruitment through non-profit patient support organisations | 3 months | Self-report questionnaire | √ | x | Not specified |
| Aguilar-Garduño, et al (2010) [56] | Mexico | case-control | 2000 - 2001 | 110 | Register of the Epidemiological Surveillance System for Neural Tube Defects | Prior to 3 months before conception (up to 5 years) | Interviewer administered questionnaire | √ | √ | Neonate |
| Bae, et al (2018) [52] | United States | prospective | 2005 - 2009 | 235 couples | Existing cohort known as the LIFE study, and couples who discontinued contraception and were trying for pregnancy | 12 months | Non-fasting serum concentrations | √ | √ | Birth |
| Cassidy, et al (1989) [57] | United States | case-control | Not specified | 81 | Not specified | At the time of conception | Case-records/telephone interview | x | √ | Not specified |
| Castro-Jiménez, et al (2011) [58] | Colombia | case-control | Jan 2000 - Mar 2005 | 170 | Cases: Colombian institutional registries  Controls: individually matched neighbourhood | 24 months | Job-exposure matrix | √ | √ | Below age 15 |
| Chiu, et al (2017) [50] | United States | prospective | 2007 - 2016 | 325 | Existing cohort known as the Environment and Reproductive Health Study | Not specified (Previous 3 months upon entry) | Self-report questionnaire | √ | x | Not specified |
| Gashaw, et al (2021) [47] | Ethiopia | case-control | Oct 2019 - Apr 2020 | 243 | Five North Shoa Zone Hospitals, Amhara Region, Ethiopia | 1 month | Self-report questionnaire | √ | x | Neonate |
| Greenop, et al (2013) [51] | Australia | case-control | Jan 2005 - Dec 2010 | 1,134 | Cases: existing cohort from the Aus-CBT study  Controls: existing cohort from the Aus-ALL study | 12 months | Self-report questionnaire and computer-assisted telephone interview | √ | √ | Age 0 to 1; age 2 to 4; age 5 to 9; age 10 to 14 |
| Heck, et al (2019) [59] | Denmark | cross-sectional | 1968 - 1974 | 217 | Danish cancer registry | 3 months | Job-exposure matrix | x | √ | Below age 20 |
| Hu, et al (2020) [49] | China | prospective | Jul 2017 - Dec 2018 | 552 | Women who underwent IVF at the Center for Reproductive Medicine | Not specified | Urine concentrations | √ | x | Not specified |
| Lacasaña, et al (2006) [44] | Mexico | case-control | Mar 2000 - Feb 2001 | 302 | Epidemiological Surveillance System of Neural Tube Defects in Mexico | Prior to 3 months preconception | Self-report questionnaire | √ | √ | Neonate |
| Ly, et al (2017) [46] | International (Vietnam, Philippines, Honduras, Morocco) | case-control | 2011 - 2015 | 626 | Cases: children recruited in Operation Smile  Controls: public hospitals in the selected countries | 2 to 8 months | Interviewer administered questionnaire | √ | √ | Below age 3 |
| Miligi, et al (2013) [18] | Italy | case-control | 1998 - 2001 | 1,824 | Cases: SETIL study (participants recruited through paediatric oncology centres)  Controls: chosen randomly from the local population in each region using the national health service records | 12 months | Interviewer administered questionnaire | √ | √ | Age 0 to 10 |
| Murphy, et al (2010) [53] | United States | prospective | 1995/96 - 1998 | 50 | Large population-based cohort, The New York Angler Cohort Study | Up to 12 months | Non-fasting serum concentrations | √ | x | Birth |
| Mustieles, et al (2018) [42] | United States | prospective | 2005 - 2016 | 536 | Environment and Reproductive Health (EARTH) Study at the Massachusetts General Hospital Fertility Centre | Not specified | Urine concentrations | √ | √ | Neonate |
| Olshan, et al (1990) [60] | United States | case-control | Jun 1984 - May 1986 | 433 | National Wilms' Tumor Study | Any time prior to pregnancy | Job-exposure matrix | x | √ | Below age 15 |
| Parodi, et al (2014) [61] | Italy | case-control | 1998 - 2001 | 1,197 | Paediatric Oncology Centres of the Italian Association of Paediatric Haematology and Oncology (AIEOP) | Not specified | Interviewer administered questionnaire | √ | x | 0 to 17 months; 18 to 59 months; > 60 months of age |
| Qu, et al (2017) [97] | China | prospective | Jan 2010 - Dec 2012 | 243,362 | Routine records (a national pre-conceptional care program) | Not specified  (‘prior to conception’) | Self-report questionnaire | √ | √ | Not specified |
| Qu, et al (2019) [19] | China | prospective | 2010 – 2013 | 230,728 | National Free Preconception Health Examination Project | Within 6 months (not specified) | Self-report questionnaire | √ | √ | Neonate |
| Robledo,[54] et al (2015) | United States | prospective | 2005 - 2009 | 234 | An existing cohort known as the Longitudinal Investigation of Fertility and the Environment (LIFE) Study | Not specified | Non-fasting serum concentrations | √ | √ | Neonate |
| Slater, et al (2011) [71] | United States and Canada | case-control | Jan 1996 - Oct 2002 (phase I); Jan 2003 - Dec 2006 (phase II) | 760 | Infants from a Children's oncology group diagnosed with leukemia | 1 month | Interviewer administered questionnaire | √ | x | Below age 1 |
| Smarr, et al (2015) [43] | United States | prospective | 2005 - 2009 | 466 couples  (233 infants) | Longitudinal Investigation of Fertility and the Environment (LIFE) Study | Not specified | Urine concentrations | √ | √ | Neonate |
| Weisskopf, et al (2005) [55] | United States | case-control | 1993 - 1995 | 143 mother-infant pairs | Cases: Licensed sport-fishing charter boat captains from the consortium states assumed to be frequent consumers of Great Lakes sport-caught fish  Controls: a random sample of the general population who were infrequent fish consumers | Not specified  (reported as 'before pregnancy') | Self-report questionnaire | √ | x | Birth |
| Weselak, et al (2008) [48] | Canada | Not specified | Jan 1990 - Dec 1993 | 3,412 | Family-run farms in Ontario, 1986 | 3 months | Self-report questionnaire | √ | √ | Newborn |
| Zhang, et al (2020) [40] | United States | prospective | Jan 2005 - Dec 2018 | 648 | Females and males in the Environment and Reproductive Health (EARTH) Study at the Massachusetts General Hospital Fertility Center and seeking fertility treatment | Not specified | Urine concentrations | √ | √ | Neonate |
| Zhang, et al (2021) [41] | United States | prospective | 2005 - 2018 | 595 | Females and males seeking infertility treatment in the Environment and Reproductive Health (EARTH) Study at the Massachusetts General Hospital Fertility Centre | Not specified  (Urine samples at entry, at each cycle of attempted pregnancy up to conception) | Urine concentrations | √ | √ | Neonate |

**Supplementary Table 6: Characteristics of included studies investigating outcomes associated with other general exposure categories (n=24)**

| **Study** | **Country** | **Study design** | **Date** | **Total participants** | **Population** | **Preconception period** | **Exposure assessment method** | **Exposure population** | | **Age of child** |
| --- | --- | --- | --- | --- | --- | --- | --- | --- | --- | --- |
|  |  |  |  |  |  |  |  | **Maternal** | **Paternal** |  |
| Bloom, et al (2015) [70] | United States | prospective | 2005 - 2009 | 235 | Longitudinal Investigation of Fertility and the Environment study. Couples planning pregnancy, committed heterosexual, females 18-40 and males 18+ without fertility issues | Not specified  (Intention to pregnancy) | Blood concentrations | √ | √ | Birth |
| Bross and Natarajan (1974) [65] | United States | case-control | 1959 - 1962 | 2,278 | Tri-State Leukemia Survey | Not specified | Not mentioned | √ | x | Age 1 to 14 |
| Bunch, et al (2009) [66] | Britain | case-control | 1952 - 1999 | Pooled analysis – 52,612 | National Registry of Childhood Tumours (NRCT) children below age 15 diagnosed with cancer, National Registry for Radiation Workers (NRRW) (mothers occupationally exposed to ionising radiation) | Not specified | National registry – The National Registry for Radiation Workers (personal monitoring device - dosemeter) | √ | x | Below age 15 |
| Castro-Jiménez, et al (2011) [58] | Colombia | case-control | Jan 2000 - Mar 2005 | 170 | Cases: Colombian institutional registries  Controls: individually matched neighbourhood | 24 months | Job-exposure matrix | √ | √ | Below age 15 |
| Chen, et al (2008) [77] | China | case-control | 2002 - 2004 | 1,995 | A project which included a cohort of pregnant women in Wuxi, China. | 12 months | Self-report questionnaire | √ | x | 28 weeks of pregnancy to 7 days after birth |
| Goel, et al (2009) [67] | United States and Canada | case-control | 1999 - 2002 | 1,021 | Newly diagnosed Wilms tumor patients less than 16 years of age, at one of the 128 participating hospitals in the United States and Canada | 12 months; 12 to 24 months | Computer-assisted telephone interview | √ | x | Below age 16 |
| Green, et al (1997) [62] | Canada | case-control | April 1979 - Dec 1986 | 928 | Population-based registry of Canadian congenital anomalies surveillance system | 6 months; 2 months | Personal monitoring device | √ | √ | Below age 1 |
| Liu, et al (2013) [74] | China | case-control | Feb 2010 - Oct 2011 | 754 | Four hospitals providing a prenatal clinic | 3 months; 4 to 6 months; 7 to 12 months | Interviewer administered questionnaire | √ | x | Not specified |
| Liu, et al (2017) [73] | China | cross-sectional | 2010 - 2012 | 213,461 | Existing cohort known as the National Pre-pregnancy Checkups Project (NPCP) | 6 months | Interviewer administered questionnaire | √ | x | 1 month of age |
| Ly, et al (2017) [46] | International (Vietnam, Philippines, Honduras, Morocco) | case-control | 2011 - 2015 | 626 | Cases: children recruited in Operation Smile  Controls: public hospitals in the selected countries | 2 to 8 months | Interviewer administered questionnaire | √ | √ | Below age 3 |
| Meinert, et al (1999) [68] | Germany | case-control | 1980 - 1994 | 4,946 | German Childhood Cancer Registry | 24 months | Self-report questionnaire and telephone interview | √ | √ | Below age 15 |
| Miligi, et al (2013) [18] | Italy | case-control | 1998 - 2001 | 1,824 | Cases: SETIL study (participants recruited through paediatric oncology centres)  Controls: chosen randomly from the local population in each region using the national health service records | 12 months | Interviewer administered questionnaire | √ | √ | Age 0 to 10 |
| Nie, et al (2013) [72] | China | case-control | Jul 2004 - Dec 2011 | 5,136 | A congenital heart disease network which included 34 hospitals | 3 months | Self-report questionnaire | √ | x | Age 1 year and below |
| Olshan, et al (1990) [60] | United States | case-control | Jun 1984 - May 1986 | 433 | National Wilms' Tumor Study | Any time prior to pregnancy | Job-exposure matrix | x | √ | Below age 15 |
| Ou Shu, et al (1999) [69] | United States | case-control | Jan 1989 - Jun 1993 | 3,828 | Children's Cancer Group | Not specified | Interviewer administered questionnaire | √ | √ | Below age 15 |
| Perez-Zaldizar, et al (2008) [78] | Mexico | case-control | 1999 - 2000 | 386 | Four public hospitals in Mexico City | 24 months | Interviewer administered questionnaire | x | √ | Age 2 to 5 |
| Perez-Zaldizar, et al (2016) [79] | Mexico | case-control | 1998 - 2013 | 564 | Population-based cancer registry maintained by the Instituto Mexicano del SeguroSocial (IMSS) and the MIGICCL. | Not specified | Interviewer administered questionnaire | √ | √ | Below age 2 |
| Qu, et al (2019) [19] | China | prospective | 2010 - 2013 | 7,299 | National Free Preconception Health Examination Project | Up to 6 months | Self-report questionnaire | √ | √ | At birth |
| Schüz, et al (2000) [20] | Germany | Case-control (pooled analysis from three studies) | 1992 - 1997 | 4,100 | (1) a case-control study on childhood cancer in the Northwestern part of Germany; (2) a case-control study on childhood leukaemia and childhood lymphoma in the vicinity of German nuclear installations and selected control regions; (3) a case-control study on childhood cancer covering the entire region of Western Germany. | 12 months | Self-report questionnaire | √ | √ | Below age 15 |
| Sever, et al (1988) [63] | United States | case-control | 1957 - 1980 | 1,649 | Hanford (place) workers exposed to whole body penetrating radiation | exposure cumulative to the conception date | Personal monitoring device | √ | √ | At birth |
| Shea, et al (1997) [64] | England | prospective | 1991 - 1992 | 7678 | Avon Longitudinal Study of Pregnancy and Childhood (ALSPAC) | 12 months | Self-report questionnaire | x | √ | Not specified |
| Slater, et al (2011) [71] | United States and Canada | case-control | Jan 1996 - Oct 2002 (phase I); Jan 2003 - Dec 2006 (phase II) | 760 | Infants from a Children's oncology group diagnosed with leukemia | 1 month | Interviewer administered questionnaire | √ | x | Below age 1 |
| Sung, et al (2009) [75] | Taiwan | Other: retrospective cohort | May 1973 - Dec 1992 | 7,202 male workers  (13,592 liveborn children) | Taiwan Bureau of Labor Insurance, with linked data from the Taiwan National Birth and Death Registry | 3 months | Government database – Bureau of Labor Insurance | x | √ | Below age 1 |
| Svanes, et al (2017) [76] | Europe | prospective | 1991 - 2012 | 24,168 | Offspring over 2 years old of previous cohort participants for RHINE III cohort study involving seven northern European centres | Up to 10 years | Self-report questionnaire | √ | √ | Below age 10 |
